# The Role of Anthocyanins, Curcumin, and Resveratrol in the Prevention and Management of Metabolic Disorders: A Systematic Review

**DOI:** 10.3390/molecules31111837

**Published:** 2026-05-26

**Authors:** Patrycja Gazda, Paweł Glibowski

**Affiliations:** Department of Biotechnology, Microbiology and Human Nutrition, Faculty of Food Sciences and Biotechnology, University of Life Sciences in Lublin, 20-950 Lublin, Poland; patrycja.gazda@up.edu.pl

**Keywords:** anthocyanins, curcumin, resveratrol, metabolic disorders, obesity, type 2 diabetes, dyslipidemia, systematic review

## Abstract

Metabolic disorders such as obesity, type 2 diabetes, and lipid disorders are major health challenges worldwide. There is increasing interest in the role of food-derived antioxidants in the context of metabolic disorders due to their documented antioxidant activity. Antioxidants such as flavonoids and polyphenols neutralize reactive oxygen species and reduce oxidative stress, which can affect cell function and metabolic processes. Anthocyanins, curcumin, and resveratrol exhibit physiological and pharmacological properties such as antioxidant, anti-inflammatory, anti-cancer, anti-obesity, and anti-diabetic effects. The main aim of this systematic review is to comprehensively evaluate and synthesize the current scientific evidence on the role of anthocyanins, curcumin, and resveratrol in the prevention and management of metabolic disorders, with a focus on obesity, type 2 diabetes, and dyslipidemia. Databases such as PubMed and Embase were searched, and the final selection included 105 studies that met the inclusion criteria. The analyzed studies demonstrated that anthocyanin supplementation (up to 320 mg/day) was associated with reductions in inflammatory markers such as IL-6 and TNF-α, improvements in HDL cholesterol, and modest reductions in HbA1c (~0.3–0.5%). Curcumin supplementation was associated with decreases in body weight (up to 0.82 kg), BMI (up to 0.30 kg/m^2^), triglycerides, total cholesterol, and fasting glucose levels. Resveratrol showed mixed but potentially beneficial effects on insulin sensitivity, oxidative stress markers, and lipid metabolism, although the clinical outcomes remained inconsistent across studies. These findings suggest that the antioxidant effects of anthocyanins, curcumin, and resveratrol may be related to their ability to suppress oxidative stress and inflammatory processes, thereby contributing to improvements in glucose and lipid metabolism. The conclusions from this analysis may contribute to a better understanding of the role of antioxidants in the management of metabolic health and indicate directions for future research in this area.

## 1. Introduction

Metabolic disorders pose a serious challenge to healthcare systems worldwide due to their significant impact on quality of life, requiring a holistic approach in the context of prevention, diagnosis, and treatment. Antioxidants (such as vitamin C, vitamin E, beta-carotene, lycopene, lutein, zeaxanthin, flavonoids, selenium, zinc, anthocyanins, curcumin, and resveratrol), which are natural bioactive compounds present in numerous plant-based foods (e.g., vegetables, fruits), play an important role in the prevention of metabolic diseases through a variety of biological mechanisms, including the modulation of oxidative stress and inflammation. Anthocyanins, curcumin, and resveratrol have documented antioxidant and anti-inflammatory effects and may have beneficial effects on metabolic disorders. The selection of these compounds was based on their extensive investigation in preclinical and clinical studies of metabolic disorders, as well as their representation of structurally distinct classes of dietary polyphenols with well-characterized biological activities. Other antioxidant classes (e.g., vitamins, minerals, and carotenoids) were excluded in accordance with the defined scope of this review, which focused specifically on plant-derived polyphenolic compounds. However, the beneficial effect of antioxidants on patients with metabolic diseases remains under investigation [1]. Obesity, defined as excessive accumulation of adipose tissue, is strongly associated with metabolic complications, including hypertension, dyslipidemia, and type 2 diabetes [2,3]. Chronic low-grade inflammation associated with obesity contributes to metabolic dysfunction through the increased production of pro-inflammatory cytokines and adipocytokines [4].

Studies show that obesity significantly increases the risk of chronic diseases, including type 2 diabetes, cardiovascular disease, hypertension, dyslipidemia, hyperuricemia, and insulin resistance [5,6]. Dietary patterns characterized by a high consumption of calorie-dense and highly processed foods contribute to excessive body weight and metabolic imbalance [7,8]. Oxidative stress is considered one of the important pathophysiological mechanisms linking obesity with chronic inflammation and metabolic disorders. Excessive production of reactive oxygen species (ROS), accompanied by impaired antioxidant defense, contributes to cellular damage and metabolic dysfunction [9,10,11].

Obesity is closely associated with dyslipidemia and insulin resistance, both of which contribute to impaired glucose and lipid metabolism [12,13]. In obese individuals, disturbances in carbohydrate metabolism may lead to prediabetes and type 2 diabetes. Worldwide, diabetes affects more than half a billion people, accounting for over 10.5% of the global adult population. Moreover, projections point to a worrisome upward trend to 12.2% (783.2 million) in 2045 [14].

This review aims to summarize current knowledge on the role of antioxidant compounds, with particular emphasis on anthocyanins, curcumin, and resveratrol, in the prevention and modulation of metabolic disorders. Another important aspect of this study is to discuss the antioxidant activity of anthocyanins, curcumin, and resveratrol in relation to selected metabolic disorders and accompanying inflammation. This may contribute to a better understanding of the mechanisms underlying their biological activity and help identify potential therapeutic targets. In addition, the observed intense increase in the incidence of metabolic diseases in the world population, with the constant search for holistic therapeutic solutions, highlights the importance of a comprehensive analysis of current knowledge on the role of antioxidants in preventing metabolic disorders. This review provides a comprehensive overview of clinical evidence concerning the potential role of these bioactive compounds in metabolic health. The main objective of this systematic review is to critically evaluate and synthesize the available scientific evidence regarding the effects of anthocyanins, curcumin, and resveratrol on the prevention and management of metabolic disorders, including obesity, type 2 diabetes, and dyslipidemia, with particular emphasis on their potential mechanisms of action related to oxidative stress and inflammation.

## 2. Methodology

**Figure 1:**
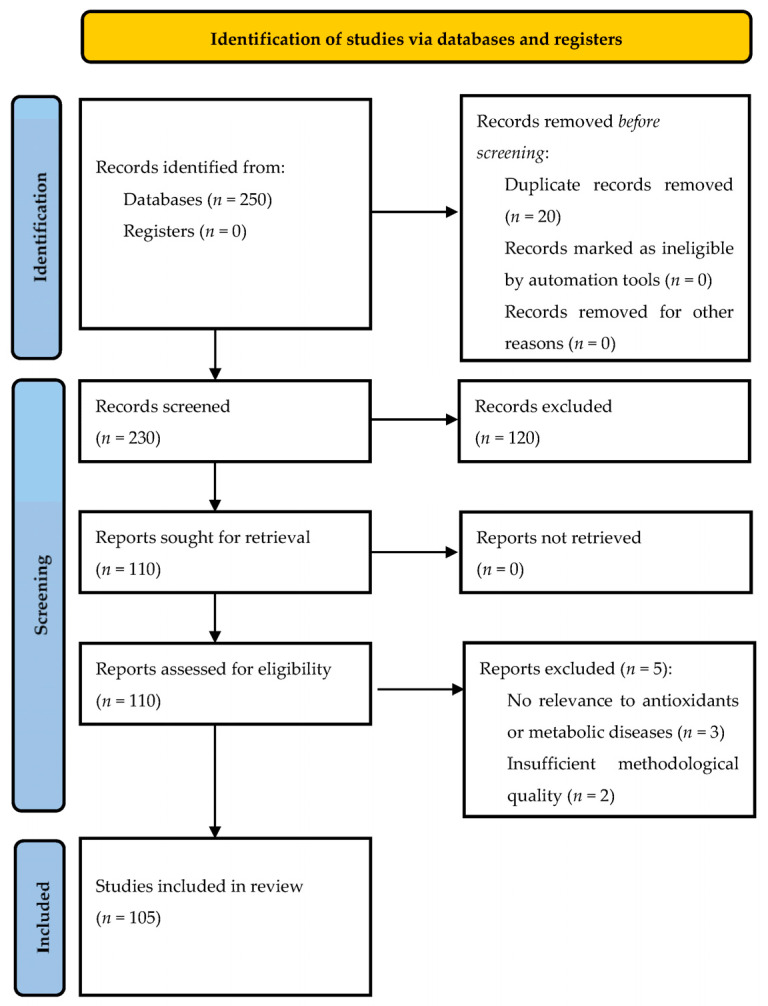
PRISMA 2020 flow diagram for new systematic reviews which included searches of databases and registers only.

This systematic review was conducted in accordance with the Preferred Reporting Items for Systematic Reviews and Meta-Analyses (PRISMA 2020) guidelines to ensure transparency and reproducibility of the methodology [15]. A systematic literature search was carried out using the following electronic databases: PubMed, Embase, ScienceDirect, Scopus, Google Scholar, and Web of Science. PubMed and Embase were selected as the primary biomedical databases due to their broad coverage of clinical and biomedical literature, while Web of Science and Scopus were included to increase the comprehensiveness of citation tracking and interdisciplinary coverage. ScienceDirect was additionally searched to identify relevant full-text articles related to nutrition, antioxidants, and metabolic disorders. Google Scholar was used as a supplementary source to *minimize* the risk of missing potentially relevant studies. The search was performed between November 2024 and January 2025 and included studies published from January 2016 onward. A comprehensive search strategy was developed using a combination of controlled vocabulary terms (e.g., MeSH terms in PubMed) and free-text keywords. The main search string applied in PubMed was as follows: (“antioxidants”[MeSH Terms] OR antioxidants) AND (“obesity” OR “dyslipidemia” OR “metabolic disorders” OR “prediabetes” OR “diabetes”) AND (“anthocyanins” OR “resveratrol” OR “curcumin” OR “oxidative stress”). The search syntax was adapted appropriately for each database (e.g., Emtree terms for Embase, TITLE-ABS-KEY fields for Scopus and Web of Science). For PubMed, Medical Subject Headings (MeSH) and Boolean operators were applied. In Embase, Emtree terms combined with free-text keywords were used. Searches in Scopus and Web of Science were conducted using TITLE-ABS-KEY and Topic field combinations, respectively. ScienceDirect and Google Scholar searches were based primarily on keyword combinations related to antioxidants and metabolic disorders, with filters applied for publication year and article relevance. Truncation symbols and database-specific operators were additionally adjusted to optimize the sensitivity and specificity of the search process. In addition, reference lists of relevant articles were manually screened to identify further eligible studies. The study selection process followed three stages: duplicate removal, title and abstract screening, and full-text assessment. Study selection was performed independently by two reviewers. In the first stage, duplicate records were removed. Duplicate identification and removal were performed manually and through reference management software by comparing titles, authors, publication years, and DOI numbers across databases. In the second stage, titles and abstracts were screened to identify potentially relevant studies. In the final stage, full-text articles were assessed for eligibility based on predefined inclusion and exclusion criteria. Any disagreements were resolved through discussion. The reasons for exclusion during full-text assessment were documented and are summarized in the PRISMA flow diagram (Figure 1). A total of 250 records were initially identified. After the removal of duplicates (*n* = 20), 230 records remained for the screening of titles and abstracts. Of these, 120 records were excluded as irrelevant or not meeting the inclusion criteria. Subsequently, 110 full-text articles were assessed for eligibility, of which 5 were excluded due to insufficient methodological quality or lack of relevance. Ultimately, 105 studies were included in the review (Figure 1). The inclusion criteria comprised studies published since 2016 that specifically addressed the role of antioxidants in metabolic disorders, including obesity, prediabetes, dyslipidemia, and diabetes. The exclusion criteria included duplicate publications, articles in languages other than English, and studies unrelated to the research question. Conference abstracts, editorials, and studies lacking sufficient methodological details were also excluded. Data extraction was conducted independently by two reviewers using a standardized data extraction form. Extracted data included study design, population characteristics, type and dose of antioxidant, duration of intervention, and reported outcomes related to metabolic parameters. Any discrepancies were resolved through discussion. All outcomes for which data were sought were predefined and included metabolic parameters (e.g., glucose levels, insulin resistance indices), inflammatory markers (e.g., CRP, cytokines), and oxidative stress indicators. All reported results compatible with each outcome domain were collected where available, including different measurement methods and time points. Additional variables extracted included study design, participant characteristics (e.g., age, sex, baseline health status), intervention characteristics (type, dose, and duration of antioxidant supplementation), and comparator details. In cases of missing or unclear data, information was interpreted based on the available descriptions in the original articles. When necessary, attempts were made to clarify unclear methodological details through additional examination of Supplementary Materials and related publications. No assumptions were made regarding unreported outcomes. The risk of bias in the included studies was assessed using established and standardized tools appropriate to the study design. For randomized controlled trials, the Cochrane Risk of Bias tool (RoB 2) was applied, while non-randomized studies were evaluated using the ROBINS-I tool. For observational studies, elements of the Newcastle–Ottawa Scale were considered. The assessment included the evaluation of selection bias, performance bias, detection bias, attrition bias, and reporting bias. Only studies with acceptable methodological quality were included to minimize systematic errors and increase the reliability of the findings. The certainty of evidence for each outcome was assessed using the GRADE (Grading of Recommendations, Assessment, Development and Evaluations) approach. The assessment considered study limitations, inconsistency of results, indirectness of evidence, imprecision, and potential publication bias. The overall certainty of evidence was classified as high, moderate, and low. Due to the heterogeneity of the study designs and reported outcomes, results were synthesized narratively. Effect measures reported in the included studies included mean differences, percentage changes, and relative risk estimates. Additionally, the risk of bias due to missing results (reporting bias) was assessed qualitatively by evaluating the consistency of reported outcomes across studies and considering the potential for selective outcome reporting. Due to the absence of a meta-analysis, no formal statistical methods (e.g., funnel plots or Egger’s test) were applied. The potential impact of reporting bias was considered during the interpretation of the findings. Studies were grouped for synthesis based on the type of antioxidant intervention, including anthocyanins, curcumin, and resveratrol. Grouping was performed by comparing study characteristics such as intervention type, study population, and reported outcomes. No formal data transformations were performed. Extracted data were reported as presented in the original studies. In cases of missing or inconsistent reporting, available data were interpreted qualitatively without imputation. The results of individual studies and syntheses were presented using structured tables and narrative descriptions to facilitate comparison across studies. Due to heterogeneity in study design, populations, interventions, and outcomes, a narrative synthesis approach was applied. A meta-analysis was not conducted as the included studies were not sufficiently comparable. Potential sources of heterogeneity, including differences in study populations, intervention dosages, duration of supplementation, and outcome assessment methods, were explored qualitatively. Sensitivity analyses were not conducted due to the narrative nature of the synthesis and the heterogeneity of the included studies. The growing interest in the role of antioxidants in metabolic disorders is reflected in the increasing number of publications indexed in the PubMed database under the search term “antioxidants and metabolic disorders”.

Furthermore, the study characteristics (study population, intervention, duration of the study, main results, conclusions) are presented in Table 1.

## 3. Oxidative Stress and Metabolic Disorders

Oxidative stress is an imbalance between reactive oxygen species (ROS) and antioxidants. Under pathological conditions, antioxidant defense systems lose their ability to detoxify these reactive species. Commonly used markers of oxidative stress include products of lipid peroxidation (e.g., malondialdehyde), protein oxidation, and DNA damage, as well as changes in antioxidant enzyme activity (e.g., superoxide dismutase, catalase, and glutathione peroxidase) [16,17]. Overproduction of reactive oxygen species leads to the development of inflammation, which is an important precursor of many diseases, including obesity, diabetes, dyslipidemia, hypertension, coronary artery disease, and other chronic conditions [1]. Excessive ROS production disrupts cellular signaling, mitochondrial activity, and insulin responsiveness, thereby contributing to metabolic dysfunction. Additionally, oxidative stress is associated with impaired glucose metabolism and lipid metabolism, circulatory and respiratory dysfunction, mitochondrial impairment, endothelial dysfunction, and chronic inflammation [18].

### 3.1. Obesity

Oxidative stress plays a key role in the pathophysiology of obesity. Obesity is often associated with chronic low-intensity inflammation that leads to the increased production of pro-inflammatory cytokines such as interleukin-6 (IL-6), tumor necrosis factor (TNF-α), and interleukin 1 (IL-1) [19]. Inflammation in obesity leads to the increased production of reactive oxygen species, which play an important role in the development of many metabolic complications of obesity [20]. Persistent ROS accumulation enhances lipid peroxidation, protein oxidation, and mitochondrial dysfunction, thereby aggravating obesity-associated metabolic complications. Inflammation associated with obesity further exacerbates oxidative stress [21]. The body’s antioxidant defense system limits the level of reactive oxygen species. ROS are generated through both enzymatic and non-enzymatic pathways. The enzymatic system includes enzymes such as mitochondrial and intracellular superoxide dismutase, transferase, catalase, and glutathione peroxidase. These enzymes neutralize ROS and protect cellular structures against oxidative damage [17]. The non-enzymatic system includes antioxidants of exogenous origin, i.e., delivered to the body with food. These include polyphenols, carotenoids, vitamins A, C, and E and minerals (selenium, copper, and zinc). This system scavenges free radicals not neutralized by enzymatic mechanisms [22]. Weakening of antioxidant defense mechanisms leads to the increased production of reactive oxygen species, which may result in mitochondrial dysfunction, manifested as a decrease in mitochondrial number and impaired oxidative protein activity due to the accumulation of ROS in cells and tissues [23]. Numerous studies show that abdominal obesity predisposes to oxidative stress, leading to multiple metabolic complications, including insulin resistance [24,25].

### 3.2. Carbohydrate Metabolism Disorders

Obesity increases the incidence of insulin resistance and its subsequent metabolic consequences, including disorders of carbohydrate metabolism. Prediabetes states are characterized by abnormal glucose homeostasis, including impaired fasting glycemia and impaired glucose tolerance (IGT), and represent a significant risk factor for the development of type 2 diabetes and its complications [26]. Commonly used diagnostic methods for both prediabetes and diabetes include fasting blood glucose measurement and the oral glucose tolerance test [27]. Diabetes is a metabolic disease that manifests itself as abnormalities in the activity and secretion of insulin, resulting in chronic hyperglycemia. There are two main types of diabetes: type 1 and type 2. Type 1 is characterized by autoimmune destruction of pancreatic β-cells in the islets of Langerhans, leading to impaired insulin production. It is most commonly diagnosed in young people. Type 2 diabetes is associated with insulin resistance and impaired insulin secretion. In this case, continuous monitoring of blood sugar levels is required to maintain glycemia stability. Treatment involves lifestyle modification, pharmacotherapy to improve insulin sensitivity, and if necessary, insulin therapy when pancreatic β-cell function declines [28]. There is growing evidence that oxidative stress plays a significant role in insulin resistance-induced chronic hyperglycemia. Increased levels of free fatty acids, adipokines, inflammatory cytokines, and impaired mitochondrial function disrupt insulin signaling, resulting in decreased glucose uptake by skeletal muscles, increased hepatic gluconeogenesis, and β-cell dysfunction, ultimately contributing to hyperglycemia [29].

Oxidative stress also plays an important role in the pathogenesis of diabetes. Hyperglycemia increases ROS production, and the resulting oxidative stress contributes to the development of many diabetes-related complications. However, the molecular mechanisms underlying oxidative stress in chronic hyperglycemia remain insufficiently understood [30].

Reactive oxygen species contribute to impaired pancreatic β-cell function. High glucose and lipid levels further exacerbate oxidative stress, negatively affecting insulin synthesis and secretion [31]. Moreover, pro-inflammatory cytokines such as interleukin-6 (IL-6) contribute to chronic low-grade inflammation, leading to endothelial dysfunction and atherosclerosis in type 2 diabetes [32]. A similar relationship is observed in insulin resistance, which disturbs the balance in glucose metabolism, consequently leading to hyperglycemia, which increases the oxidative stress of cells. Lipid metabolism in the body is also disturbed, which contributes to dyslipidemia [33].

### 3.3. Dyslipidemia

Dyslipidemia is a key component of metabolic disorders, characterized by abnormal lipid metabolism that contributes to insulin resistance, chronic inflammation, and increased cardiovascular risk. It is characterized by elevated triglycerides and free fatty acids, reduced HDL cholesterol levels, and increased LDL cholesterol levels acids [34]. Due to the multifactorial etiology of dyslipidemia, including lifestyle, diet, genetic factors, and obesity, its management requires an interdisciplinary approach [35]. Dyslipidemia is associated with the dysfunction of adipocytes and hepatocytes [36]. In obesity, adipocyte hypertrophy leads to the increased production of pro-inflammatory adipokines and free fatty acids, promoting inflammation, dyslipidemia, and ectopic fat deposition. Excess LDL cholesterol may be taken up by macrophages and accumulate in the vascular wall, contributing to atherosclerosis. This suggests that dyslipidemia plays a key role as a risk factor for cardiovascular disease [37]. Oxidative stress and inflammation are key contributors to cardiovascular disease. Increased ROS production promotes dyslipidemia and cardiovascular complications. Excess free radicals accelerate the formation of atherosclerosis plaques [38]. Oxidative stress also leads to endothelial dysfunction. Reactive oxygen species and cytokines enhance the synthesis of inflammatory mediators [39]. An imbalance between ROS production and antioxidant defense contributes to vascular damage [40].

## 4. Antioxidants in the Therapy of Metabolic Disorders

Antioxidants are substances that inhibit oxidation processes in the body, neutralizing free radicals [41]. They play a key role in the prevention of many diseases. As described above, oxidative stress resulting from the excessive production of reactive oxygen species (ROS) is a major contributor to numerous health problems, including cardiovascular disease, type 2 diabetes, certain types of cancer, and aging [42,43]. Antioxidants alleviate oxidative stress and the associated inflammation. Fruits and vegetables exhibit antioxidant activity due to their high content of bioactive compounds such as carotenoids and flavonoids, and other polyphenols [44]. These compounds reduce oxidative stress and inflammation. Increased dietary intake of these biologically active compounds, both in the form of fresh fruits and vegetables and their processed products, is associated with a reduced incidence of many chronic diseases [45]. Multiple studies show that antioxidants possess anti-inflammatory, antioxidant, immunomodulating, anti-cancer, and anti-obesity properties [46].

### 4.1. Anthocyanins

#### 4.1.1. Antioxidant, Anti-Inflammatory, and Metabolic Mechanisms of Anthocyanins

**Figure 2:**
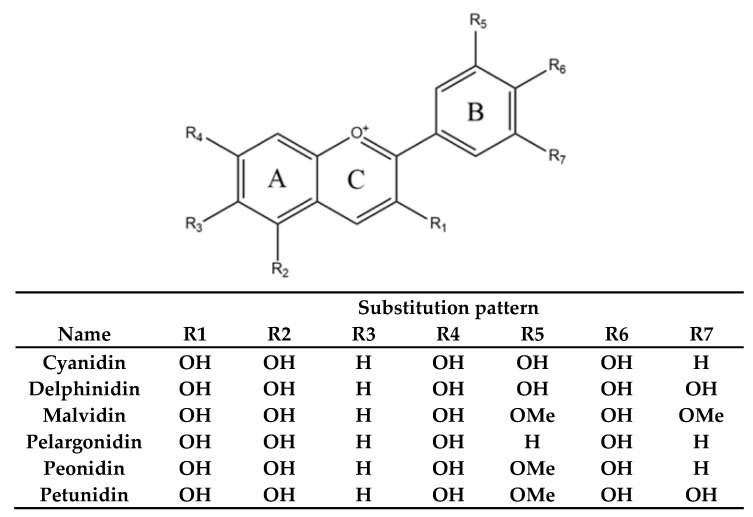
Chemical scaffold of anthocyanin compounds and their relative substituents. A: benzene ring, B: benzene ring, C: heterocyclic pyran ring [48].

Anthocyanins are water-soluble plant pigments belonging to the flavonoid group, occurring in the form of glycosides and responsible for the red, blue, and purple color of many plant tissues [47]. The anthocyanidin skeleton is associated with one to three sugar moieties, forming anthocyanins. Their chemical structure is based on the flavylium backbone (2-phenylbenzopyrylium), with variation arising from glycosylation and hydroxylation patterns. Anthocyanidins are the aglycone forms of anthocyanins, whereas anthocyanins are glycosylated derivatives of anthocyanidins. In most cases, anthocyanidins are bounded with sugar moieties, forming the corresponding anthocyanins. This glycosylation process occurs enzymatically through the attachment of sugar moieties at the 3 and/or 5 positions. Several hundred different anthocyanins have been discovered so far, but only six types are commonly found in food. The most common anthocyanins are cyanidin, delphinidin, malvidin, pelargonidin, peonidin, and petunidin. Representative chemical structures of these compounds and their characteristic substituents are presented in Figure 2 [48,49,50].

**Figure 3:**
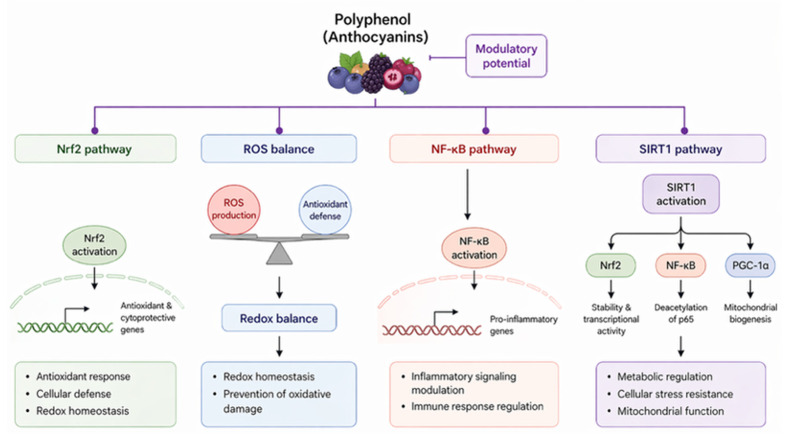
Polyphenol (anthocyanins) modulation of Nrf2 (nuclear factor erythroid 2-related factor 2), NF-κB (nuclear factor kappa-light-chain-enhancer of activated B cells), ROS (reactive oxygen species), and SIRT1 (sirtuin 1) signaling pathways.

Anthocyanins provide antioxidant protection in the pathogenesis of many obesity-related chronic diseases such as cardiovascular disease, carbohydrate metabolism disorders including diabetes, and lipid disorders. Their biological activity is mainly associated with the modulation of signaling pathways involved in inflammation, oxidative balance, and metabolic regulation [51]. Anthocyanins may be consumed either as natural components of anthocyanin-rich foods, particularly berries (e.g., blueberries, blackcurrants), or as purified extracts and dietary supplements standardized for anthocyanin content. These forms may differ in bioavailability, composition, and physiological effects. Although anthocyanins exhibit antioxidant properties in vitro, their in vivo effects are largely mediated through the modulation of cellular signaling pathways rather than the direct scavenging of free radicals. This includes the activation of endogenous antioxidant systems and regulation of redox-sensitive transcription factors [52]. The biological activity of anthocyanins is closely related to their absorption and metabolism. These compounds are absorbed in limited amounts, resulting in relatively low plasma concentrations. Absorption occurs in both the stomach and the small intestine. In the stomach, anthocyanins are partially released from food under acidic conditions, while in the small intestine, these compounds are absorbed into the bloodstream. A significant proportion of anthocyanins undergoes partial metabolism to glucuronide derivatives and sulfate conjugates, which are more water-soluble forms. Anthocyanin metabolites, such as glucuronides and sulfo-conjugates, can be present in urine for up to 24 h after the consumption of these compounds [49]. Despite limited bioavailability, these compounds and their metabolites exert significant biological effects, including antioxidant activity, modulation of inflammation, and cardiovascular protection. Anthocyanins may indirectly contribute to the reduction in oxidative stress by regulating ROS-related pathways and upregulating endogenous antioxidant systems [53]. They also modulate signaling pathways, including the activation of PPAR-γ and inhibition of NF-κB, resulting in reduced expression of pro-inflammatory cytokines such as IL-1, IL-6, and TNF-α (Figure 3) [54,55].

NF-κB is a key transcription regulator that plays a significant role in immune and inflammatory responses, as well as in cell proliferation and differentiation processes. NF-κB is a central mediator activated in response to various stimuli, such as cytokines (TNF-α, IL-1β), reactive oxygen species (ROS), bacterial and viral infections, and DNA damage, which leads to its translocation to the cell nucleus and the regulation of gene expression related to immune response, inflammation, apoptosis, and other cellular processes. SIRT1 (sirtuin 1), an enzyme protein that plays a key role in regulating metabolism, DNA repair processes, and anti-aging processes, and its activation is supported by anthocyanins present in fruits, which may contribute to protecting cells from oxidative stress. Anthocyanins, thanks to their antioxidant and immunomodulatory properties, can modulate NF-κB activity, which has therapeutic potential in inflammatory conditions, autoimmune diseases, and in regulating the body’s immune response. The transcription factor Nrf2 (nuclear factor erythroid 2-related factor 2) plays a key role in regulating the antioxidant response and protecting cells from oxidative stress [56,57]. Furthermore, anthocyanins, as antioxidants, can stimulate the expression of (PPAR-γ), which leads to adipocyte differentiation through the regulation of genes related to adipogenesis and the reduction in inflammation [58]. According to scientific research, anthocyanins have a significant antioxidant capacity, neutralize reactive oxygen species, support the production of antioxidant enzymes, such as glutathione, superoxide dismutase, catalase, and increase the expression of the nuclear erythroid 2 factor, which plays a key role in regulating antioxidant activity. Anthocyanins can modulate the activity of nuclear factors, such as erythrocyte nuclear factor 2, which translates to the increased antioxidant capacity of cells, especially erythrocytes. Such a mechanism is important for maintaining the body’s oxidative homeostasis [52].

#### 4.1.2. Human Clinical Trials on Anthocyanins

Numerous studies have demonstrated the beneficial effects of anthocyanins on metabolic health. Consumption of anthocyanin-rich foods is associated with reduced weight gain and improved metabolic parameters. However, these effects are often modest and may be transient, and their clinical significance remains limited. Current evidence suggests that anthocyanins may support metabolic health, but they should be considered as an adjunct to lifestyle interventions rather than as a primary therapeutic strategy (Table 2).

##### Weight Control and Obesity-Related Outcomes

Bertoia et al. [59] examined the impact of flavonoid intake, including flavonoids, flavonols, flavonones, flavon-3-ols, anthocyanins and flavonoid polymers, on weight control in 20,525 healthcare workers aged 40–75 years (the study started in 1986), 39,423 nurses aged 30–55 years (a study began in 1976), and 64,138 young nurses aged 25–42 years (a study begun in 1989). The participants’ intake of flavonoids was assessed using a semi-quantitative food frequency questionnaire, which was collected every 4 years. The median intake of flavonoids was 247 mg for nurses aged 30–55 years, 236 mg for 25–42 years, and 224 mg for healthcare professionals. The results of the aforementioned three cohort studies show that the consumption of foods rich in anthocyanins, flavonoids (including proanthocyanidins), and flavonoids is associated with less weight gain at four-year intervals. However, these observational findings do not establish causality and may be influenced by residual confounding factors such as overall diet quality and lifestyle behaviors. Moreover, although statistically significant, the observed differences in weight gain were relatively small and are of limited clinical relevance. These findings mainly reflect the long-term effects of the habitual intake of anthocyanin-rich foods rather than purified supplementation [59]. Similar associations were observed in a meta-analysis by Park et al. [60], which showed that purified anthocyanin supplementation up to 300 mg/day for four weeks resulted in reductions in BMI and body weight compared to the results of randomized controlled trials with a higher dose and longer treatment duration. Nevertheless, the included trials were characterized by relatively short intervention periods, small sample sizes, and heterogeneity in study design and populations, which limits the generalizability of the findings. However, these effects are generally transient, and the magnitude of weight loss (often less than 1 kg) may not meet the thresholds for clinical significance in weight management guidelines. Current guidelines prioritize sustained lifestyle modifications, including overall calorie reduction and physical activity, with dietary antioxidants playing a supportive, rather than primary, role. As it looks like the effect is only temporary, there is a need for further research on the dose- or period-dependent effects of anthocyanin supplements on various obesity biomarkers such as adipocytokines [60].

##### Inflammation and Cardiovascular Risk Factors

Anthocyanins can alleviate the consequences of poor dietary choices. A randomized, placebo-controlled cross-sectional study on the effect of cyanidine-rich and delfinidine-rich extract supplementation on inflammatory and metabolic responses following the consumption of a high-fat meal in healthy subjects demonstrated a beneficial effect of anthocyanins that resulted in a reduction in inflammation and other post-meal metabolic changes associated with the consumption of this meal. The study consisted of 25 study participants consuming a meal with a total energy content of 1026 kcal (62% of the energy came from fat, 25% from carbohydrates and 13% from protein), with simultaneous supplementation of a cyanidine-rich extract and delfinidine-rich extract (total anthocyanins were 320.4 mg, of which 150 mg was from blueberry extract, 230 mg from blackcurrant extract and 620 mg from black cranberry extract). Importantly, this study assessed acute postprandial effects occurring within hours after anthocyanin intake rather than long-term metabolic adaptations. Blood biochemical tests were conducted before the meal and after 30 min, 1, 2, 3, and 5 h after the extract was consumed. The results of the study suggest that eating foods rich in anthocyanins may offset the adverse effects of eating unhealthy meals. However, the small sample size, short-term postprandial design, and inclusion of healthy participants limit the ability to extrapolate these findings to long-term clinical outcomes or populations with metabolic disorders [61]. In contrast, Curtis et al. [62] evaluated the long-term consumption of anthocyanin-rich blueberry products over six months and observed improvements in HDL cholesterol, endothelial function, and arterial stiffness. While no changes were observed in insulin resistance or blood pressure; similarly, half a cup per day had no effect on the biomarkers. However, these findings should be interpreted with caution, as the study was conducted in a relatively small and potentially heterogeneous population with a limited intervention scope, and the lack of consistent effects across all measured parameters suggests variability in response and the possible influence of confounding factors [62]. Similarly, Zhang et al. [52] investigated the effects of chronic purified anthocyanin supplementation (40–320 mg/day) for 12 weeks in 169 individuals with dyslipidemia and demonstrated dose-dependent reductions in IL-6 and TNF-α concentrations. Anthocyanin supplementation (320 mg/day) for six weeks significantly reduced the oxidative response of total dysmutase. After 12 weeks, anthocyanin supplements at 40 mg/day moderately reduced the serum IL-6 and TNF-α concentrations, while a twofold higher dose resulted in a significant reduction in IL-6 and TNF-α levels. Supplementation at 80 mg/day showed significant improvement in reducing inflammatory marker activity. The results of the study showed that anthocyanin supplement in participants with dyslipidemia for 12 weeks significantly reduced the dose dependence on the inflammatory markers. Despite these findings, the study duration was relatively short and the population was specific (individuals with dyslipidemia), which may limit broader applicability [52]. However, another study with a similar dose and conducted by Estevez-Santiago et al. [66] showed that 8-month anthocyanin supplementation at a dose of 60 mg/day did not improve any of the plasma CRP levels, IL-6, in postmenopausal women, highlighting inconsistencies between studies that may result from differences in study design, dose, duration, and participant characteristics. Current guidelines (e.g., ESC/EAS guidelines on dyslipidemia) emphasize lipid-lowering therapies and lifestyle changes, with dietary antioxidants considered as adjuncts rather than primary interventions [66].

##### Glycemic Control and Diabetes Prevention

Three cohort studies involving 200,894 participants and 12,611 cases of type 2 diabetes found that dietary anthocyanin consumption was associated with a 15% reduction in the risk of developing the disease. Additionally, five cohort studies with 194,019 participants and 13,013 cases reported that berry consumption was linked to an 18% reduction in type 2 diabetes risk. Overall, both dietary anthocyanins and berries appear to be associated with a decreased risk of developing type 2 diabetes. Increasing dietary anthocyanins by 7.5 mg/day or increasing berry intake by 17 g/day resulted in a 5% reduction in risk of type 2 diabetes. However, these results are based on observational cohort studies and may be subject to confounding factors and measurement bias related to dietary assessment [63]. Fallah et al. [64] showed that the consumption of anthocyanins for longer than eight weeks at doses higher than 300 mg/day reduced the fasting blood sugar levels, glycated hemoglobin, and HOMA-IR in subjects with type 2 diabetes and HOMA-RI in participants with obesity. Although variability in study populations and intervention protocols should be considered when interpreting these results [64]. Similar associations were observed by Yang et al. [65] in a 12-week randomized, controlled trial among Chinese adults with pre-existing or early untreated diabetes. The study showed that purified anthocyanins (320 mg/day) had a beneficial effect on glycemia control, in particular, lowering glycated hemoglobin and lipid profile (lowering LDL cholesterol, apolipoprotein A and apolipoprotein B). Notably, reductions in HbA1c of approximately 0.3–0.5% are generally considered clinically meaningful in diabetes management, as per the ADA guidelines, which recommend lowering HbA1c by at least 0.5% when possible to reduce complication risk. In the cited studies, the observed HbA1c reductions approached these thresholds, suggesting potential benefit, but longer-term studies are needed to confirm whether such improvements sustain and translate into reduced diabetic complications. Additionally, the relatively short duration (12 weeks) and specific study population limit conclusions regarding long-term efficacy and broader clinical applicability. Further large-scale and long-term randomized trials are required to confirm these effects and their clinical relevance [65].

##### Safety and Dosage Considerations

While some studies indicate benefits at doses around 320 mg/day, others (e.g., Estevez-Santiago et al. [66] found no effect at similar doses after longer periods. Variability in response underscores the importance of dose and duration, and further research is needed to establish optimal dosing regimens. Currently, no specific clinical guidelines recommend anthocyanin supplementation, but encouraging the consumption of flavonoid-rich fruits aligns with the general dietary recommendations from the ADA, ESC, and WHO, which promote high fruit and vegetable intake for cardiovascular and metabolic health [66].

### 4.2. Curcumin

#### 4.2.1. Antioxidant, Anti-Inflammatory, and Metabolic Mechanisms of Curcumin

**Figure 4:**
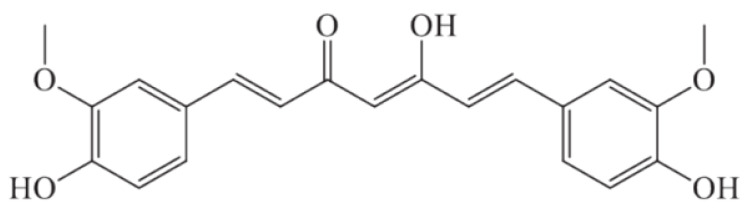
The chemical structure of curcumin [67].

Curcumin is a bioactive compound derived from *Curcuma longa* L. (Figure 4) and exhibits strong antioxidant and anti-inflammatory properties [67,68]. Its activity in vivo is mainly associated with the regulation of inflammatory and redox-sensitive pathways, including NF-κB and Nrf2 signaling. Despite broad preclinical efficacy, its clinical translation is fundamentally constrained by unfavorable pharmacokinetics, including extremely low oral bioavailability, poor aqueous solubility, rapid intestinal and hepatic metabolism, and fast systemic clearance, resulting in limited plasma exposure after oral administration. Importantly, the biological activity of curcumin in vivo is strongly dependent on its formulation, which directly determines its absorption and systemic availability. Native (standard) curcumin exhibits particularly low bioavailability due to poor solubility and rapid metabolism. To overcome these limitations, several advanced delivery systems have been developed, including phytosome complexes (e.g., curcumin–phospholipid complexes), nanoparticle-based formulations, liposomal encapsulation, and adjuvant-enhanced preparations such as curcumin combined with piperine. Piperine, an alkaloid from Piper nigrum, can inhibit hepatic and intestinal glucuronidation, thereby significantly increasing the curcumin plasma concentrations. Similarly, phytosome and nanoparticle formulations improve intestinal absorption and systemic exposure by enhancing solubility and protecting curcumin from premature metabolic degradation. Consequently, the observed therapeutic efficacy of curcumin in preclinical and clinical studies is highly formulation-dependent, with enhanced formulations generally demonstrating superior pharmacokinetic profiles and more consistent biological effects compared to unformulated curcumin. Curcumin demonstrates therapeutic potential due to its anti-inflammatory, anti-aging, anti-diabetic, and anti-cancer effects. Curcumin is poorly soluble at room temperature in aqueous solutions with neutral and acidic pH. Curcumin has the ability to form stable complexes with heavy metals such as copper, chromium, arsenic, mercury, lead, and cadmium, thereby potentially contributing to reduced oxidative stress [69].

**Figure 5:**
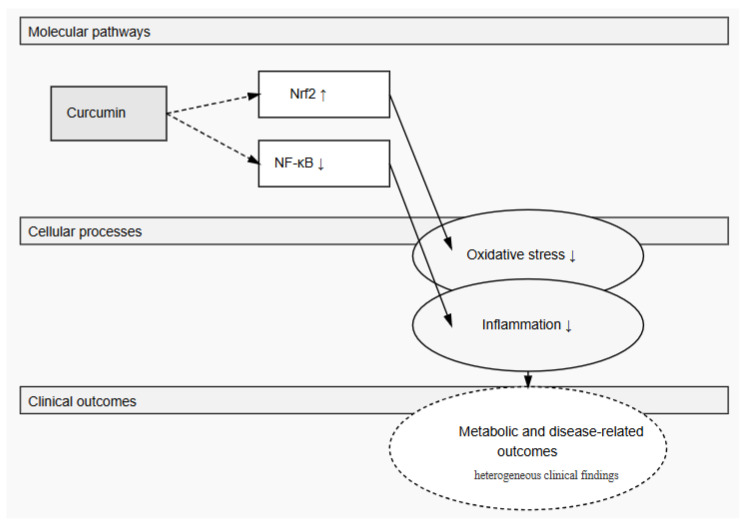
Curcumin-mediated regulation of oxidative stress, inflammation, and metabolic dysfunction (NF-κB—nuclear factor kappa-light-chain-enhancer of activated B cells, Nrf2—nuclear factor erythroid 2-related factor 2, SIRT1—sirtuin 1). ↑ increase, ↓ decrease.

Turmeric contains a polyphenol compound called curcumin, which has a number of health-promoting properties, including anti-inflammatory, anti-proliferative, and antioxidant effects. Curcumin has a multifaceted effect, thanks to its ability to modulate signaling pathways related to inflammation, oxidative stress, and proliferation processes [70]. It modulates key signaling pathways, including NF-κB, leading to the reduced expression of pro-inflammatory cytokines (TNF-α, IL-1β, IL-6), COX-2, and iNOS (Figure 5) [71].

By reducing oxidative stress and inflammation, curcumin improves insulin signaling and increases insulin sensitivity. Curcumin modulates key pathways, such as NF-κB, and reduces IL-6 and TNF-α signaling, which leads to improved glucose metabolism [72]. Additionally, in the context of insulin resistance, scientific evidence has shown that curcumin increases the phosphorylation of insulin receptor substrate (IRS)-1 and Akt, thereby increasing the sensitivity of cells to insulin. In addition, curcumin alleviated depressive behaviors and reversed metabolic disorders caused by chronic, mild stress. This indicates the potential of curcumin as a therapeutic agent for modulating both mood and metabolic dysfunction resulting from insulin resistance [73]. Additionally, a meta-analysis by Wang et al. [74] showed that in pancreatic β cells (RINm5F), curcumin prevents desipramine-induced apoptosis (belonging to TLPD) by inhibiting the PI3K/AKT/FOXO1 pathway and disrupting AKAP150/PKA/PP2B interaction. The findings suggest curcumin as a potential adjuvant therapy that could preserve β-cell function and improve insulin regulation during antidepressant treatment [74]. It is also worth noting that curcumin reduces the concentration of circulating MDA (malondialdehyde) and increases the activity of SOD (superoxide dismutase) in serum. A reduction in the concentration of circulating MDA indicates a decrease in lipid peroxidation, i.e., protection of the lipid components of cell membranes and lipoproteins from oxidative damage. MDA is the end product of lipid peroxidation and is often used as a marker of oxidative stress. A lower MDA level in the serum suggests that curcumin may reduce oxidative damage or enhance endogenous antioxidant defenses. On the other hand, increased SOD activity in serum indicates a strengthening of the first line of antioxidant defense. SOD converts the superoxide anion O_2_− into molecular hydrogen peroxide (H_2_O_2_), which is then broken down by other antioxidant enzymes, such as catalase or glutathione peroxidase. However, further research on curcumin in various populations is necessary, taking into account many redox state biomarkers [75].

#### 4.2.2. Clinical Trials with Humans of Curcumin

Clinical studies suggest that curcumin supplementation may improve anthropometric and metabolic parameters, including body weight, BMI, and lipid profile. However, the magnitude of these effects is generally modest, and their clinical relevance requires further investigation. Curcumin has also shown beneficial effects on glycemic control, including reductions in fasting glucose and HbA1c. However, these improvements are typically smaller than those achieved with standard pharmacological treatments. Therefore, curcumin should be considered as an adjunct therapy rather than a standalone treatment for metabolic disorders [76]. Results of some of these studies are presented in Table 3.

A meta-analysis by Dehzad et al. [77] showed the positive effect of curcumin supplementation on anthropometric indicators of obesity, leptin, and adiponectin. Sixty randomized controlled clinical trials with a total number of 3691 participants were included in the analysis. The results of the studies report that curcumin supplementation significantly reduced body weight (on average by 0.82 kg), body mass index (on average by 0.30 kg/m^2^), waist circumference (on average by 1.31 cm), body fat percentage, leptin (on average by 4.46 ng/mL) and increased adiponectin [77]. These changes are consistent with a beneficial trend in anthropometric parameters; however, their clinical significance warrants further exploration, especially in relation to established guidelines that emphasize substantial weight loss (>5%) for meaningful health benefits. However, the magnitude of these changes was relatively small and may not translate into clinically meaningful outcomes according to the current guidelines. Similarly, in a study conducted by Unhapipatpong et al. [78], curcumin supplementation in adults with obesity and diabetes significantly reduced the BMI, body weight, and waist circumference with mean differences of (~0.24 kg/m^2^), (~0.59 kg), and (~1.31 cm), respectively. The results of the study suggest that curcumin supplementation has a positive effect on anthropometric parameters. However, the observed effects were insignificant, and the specific factors of the study, such as the sample size and population characteristics, should be taken into account [78].

In terms of metabolic parameters, Sangouni et al. [79] conducted a randomized clinical trial in 88 patients with metabolic syndrome who were randomly assigned to four groups supplementing with curcumin and/or supplementing with coenzyme Q10 for 12 weeks. A significant reduction in triglycerides (from 224.7 ± 66.9 to 166.7 ± 72.8 mg/dL), total cholesterol (from 217.4 ± 37.4 to 186.5 ± 30.4 mg/dL), LDL cholesterol (from 89.4 ± 6.5 to 75.3 ± 5.4 mg/dL), and an improvement in HDL cholesterol (from 32.0 ± 5.6 to 42.0 ± 5.9 mg/dL) was observed in the curcumin supplementation group compared to three other groups. In contrast, no significant differences were observed between the four groups in terms of systolic and systolic pressure, fasting plasma glucose concentration, waist circumference, BMI, and body weight. The results of the study showed that curcumin improved the indicators of dyslipidemia, but had no significant effect on anthropometric indicators, glycemia control, and hypertension, although the relatively small sample size and short intervention period (12 weeks) limit the strength of the conclusions [79].

Conversely, Hellmann et al. [80] evaluated the effect of curcumin treatment, in the form of 200 mg lecithin tablets twice daily for 6 weeks, on liver fat content in 37 obese subjects. Curcumin supplementation caused slight reductions in fasting plasma glucose, triglycerides, and gamma-glutamyltransferase, but these differences were not statistically significant, except for gamma-glutamylotransferase. The results indicate that compared to the placebo, curcumin treatment for six weeks had no significant effect on the liver fat content of obese subjects with initially mild steatosis. This study was limited by a small sample size and short duration, which may have reduced the ability to detect significant effects. This underscores the need for longer-term studies or higher doses to assess the potential benefits on liver health [80].

In patients with type 2 diabetes, Yaikwawong et al. [81] demonstrated that curcumin extract (1500 mg/day) improved beta cell function in 272 obese patients with type 2 diabetes. Patients taking curcumin demonstrated significant reductions in fasting blood glucose (from 123.65 to 115.49 mg/dL), insulin (from 17.47 to 16.05 uU/mL), glycated hemoglobin (from 6.28 to 6.12%), lower levels of HOMA-IR (from 5.38 to 4.86), leptin (from 13.84 to 9.42 ug/mL) as well as higher levels of adiponectin (from 8.75 to 14.51 ug/mL) and improved β-cell function. Curcumin supplementation improves glycemia control, thereby reducing impaired glucose metabolism. Despite the relatively large sample size, the clinical significance of the observed changes remains modest and requires confirmation in long-term studies [81]. Notably, the HbA1c reduction (~0.16%) approaches the threshold for clinical relevance, as reductions of ≥0.5% are generally considered meaningful in diabetes management [83]. While promising, these results suggest that curcumin may serve as an adjunct rather than as a primary therapeutic agent for glycemic control. Similarly, a clinical study conducted by Karandish et al. [82] among 84 subjects with pre-diabetes showed that supplementation with curcumin (500 mg) and zinc (30 mg) or zinc alone (30 mg) for 90 days resulted in a reduction in BMI (~1.2 kg/m^2^) compared to the placebo. Additionally, the groups treated with curcumin (500 mg), zinc (30 mg), and zinc and curcumin showed improvements in fasting glycemia, glycated hemoglobin, and insulin compared to the placebo group. The results of the study suggest that curcumin and zinc supplementation have a beneficial effect on glycemia [82]. The results of clinical trials indicate that curcumin has significant potential in treating metabolic disorders; however, further research is necessary to determine the optimal dosage, administration methods, long-term safety, and potential side effects to maximize its therapeutic benefits. Current clinical practice guidelines, such as those from the American Diabetes Association (ADA) and European Society of Cardiology (ESC), suggest that meaningful clinical improvements—such as reductions in HbA1c (>0.5%), weight loss (>5%), and lipid profile enhancements—are typically achieved through larger changes associated with established lifestyle modifications and pharmacotherapy. Consequently, curcumin is likely best considered as an adjunct to these conventional treatments rather than as standalone therapy. While its short-term safety profile appears acceptable, additional studies are essential to establish appropriate dosing, long-term safety, and any potential adverse effects to optimize its use in managing metabolic disorders [83].

### 4.3. Resveratrol

#### 4.3.1. Antioxidant, Anti-Inflammatory, and Metabolic Mechanisms of Resveratrol

**Figure 6:**
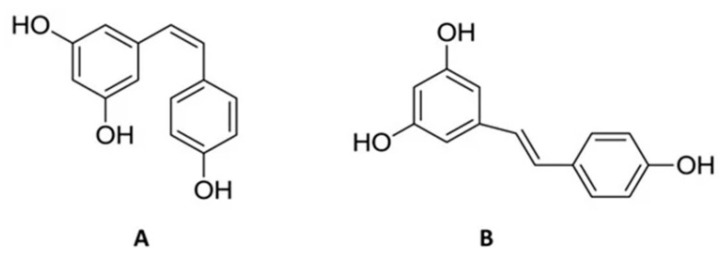
The chemical structure of cis-resveratrol (**A**) and trans-resveratrol (**B**) [85].

Resveratrol is a polyphenolic compound belonging to the stilbene group, known for its antioxidant properties. In vivo, its biological effects are predominantly linked to the regulation of inflammatory signaling and mitochondrial function [84]. This compound exists in two isomeric forms, cis-resveratrol and trans-resveratrol, with trans-resveratrol being the predominant naturally occurring and biologically most active form in plants and most experimental and clinical studies. The cis-resveratrol form is more sensitive to light, is unstable, and poorly soluble in water, dissolving mainly in solvents such as dimethyl sulfoxide (DMSO) and ethanol (Figure 6) [85].

Experimental studies suggest that resveratrol may influence glucose metabolism, inflammatory pathways, and mitochondrial activity. However, much of this evidence derives from in vitro and preclinical studies, with limited direct translational relevance to human outcomes. Resveratrol alleviates oxidative stress, inhibits inflammation, and supports mitochondrial function [86]. Studies have also shown its protective effects on the cardiovascular system, including inhibiting the oxidation of low-density lipoproteins (LDL), as well as reducing lipid peroxidation. Additionally, resveratrol treatment alleviated mitochondrial oxidative stress caused by high glucose levels. Resveratrol, through its influence on various metabolic pathways, can support the therapy of metabolic disorders associated with impaired glucose and lipid metabolism, although clinical confirmation remains inconsistent [87,88,89,90].

**Figure 7:**
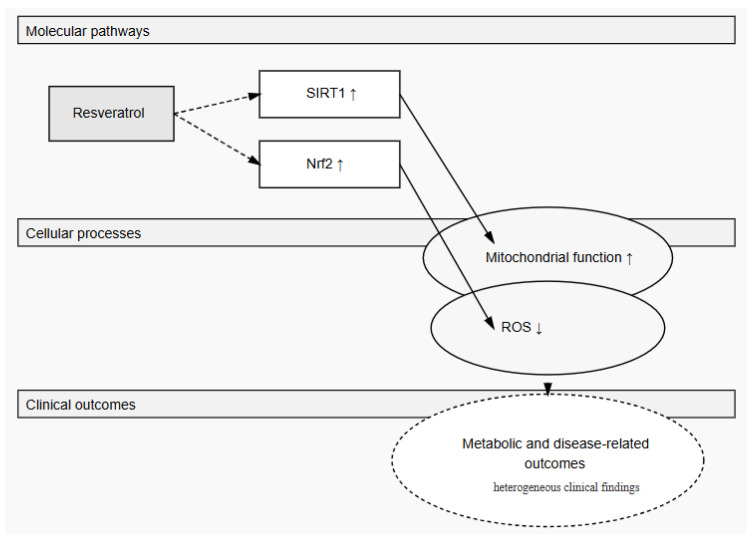
Mechanisms of resveratrol action in oxidative stress, metabolism, and cardiovascular protection (Nrf2—nuclear factor erythroid 2-related factor 2, SIRT1—sirtuin 1, ROS—reactive oxygen species). ↑ increase, ↓ decrease.

Resveratrol is a natural stilbenoid that acts on many molecular pathways, including activating SIRT1 (primarily reported for trans-resveratrol), which leads to the deacetylation of regulatory proteins and increased PGC-1α activity, and interacts with AMPK signaling pathways, which promotes energy metabolism and improves insulin sensitivity, affecting glucose metabolism regulation (Figure 7) [91].

Resveratrol exerts antioxidant effects mainly by activating endogenous defense systems (e.g., Nrf2) and modulating redox-sensitive signaling pathways, which contributes to the reduction in oxidative stress and cellular damage [92,93]. Experimental data also indicate reductions in inflammatory mediators and lipid peroxidation markers following resveratrol administration. Nevertheless, these mechanistic findings are not consistently reflected in clinical outcomes. Resveratrol may also reduce the activity of phospholipase A2 and reduce the expression of strong inflammatory factors, including prostaglandins and lymphotoxins [94]. Resveratrol reduces oxidative stress. Among the possible mechanisms is the role of H3K56ac, which has been linked to the activation of genes related to the lack of redox signal balance. It is indicated that resveratrol has the potential to induce antioxidant cytoprotection in endothelial cells by modulating the expression of SIRT1. Importantly, the majority of in vitro, animal, and clinical studies have investigated trans-resveratrol, which is considered the pharmacologically active isomer, whereas cis-resveratrol is rarely studied due to its instability and low abundance. Although numerous studies suggest that resveratrol may exert beneficial effects on inflammation, oxidative stress, and metabolic disorders, these findings are largely derived from preclinical or small-scale clinical studies characterized by substantial heterogeneity in study design (e.g., dose, duration, and population) [95].

#### 4.3.2. Clinical Trials with Humans of Resveratrol

Clinical studies on resveratrol supplementation show mixed results. Some studies demonstrate modest improvements in body weight, lipid profile, and inflammatory markers, while others show no significant effects. Interpretation of these findings is complicated by substantial heterogeneity in study design, including differences in dosage (ranging from <100 mg to >1500 mg/day), intervention duration, and population characteristics. Current evidence suggests limited and inconsistent clinical efficacy, highlighting the need for larger and longer-term randomized trials. Results of some of these studies are presented in Table 4.

The meta-analysis by Mousavi et al. [96] showed a statistically significant reduction in body weight and BMI in obese adults taking resveratrol. The results of the study showed a significant reduction in body weight (weighted mean difference: −0.51 kg, *p* = 0.02) and BMI (weighted mean difference: −0.17 kg/m^2^, *p* = 0.02) in obese adults taking resveratrol at a dose of <500 mg for at least 3 months. In contrast, no significant effect of resveratrol-supplementation on body fat was observed. However, the magnitude of these changes was small and of questionable clinical relevance [96]. Similar findings were reported by Tabrizi et al. [97], where reductions in anthropometric parameters were statistically significant but unlikely to meet the thresholds for clinically meaningful improvement (e.g., >5–10% weight loss) [83,97].

Additionally, with regard to inflammatory markers, De Ligt et al. [98] observed decreased expression of angiotensin-converting enzyme 2 (ACE2) and leptin in adipose tissue after 30 days of 150 mg/day resveratrol in obese men, indicating potential anti-inflammatory effects. Resveratrol supplementation reduced the activity of the angiotensin 2 converting enzyme (on average by about 40%) and the proinflammatory adipokine, leptin (on average by approximately 30%). While biochemical changes are promising, their direct translation into clinical outcomes remains unclear. The short intervention period and small sample size limit conclusions regarding long-term clinical outcomes. No established guidelines recommend resveratrol specifically for modulating inflammatory markers in obesity [98].

Another study evaluated the effect of oral resveratrol intake in combination with changes in diet and exercise on anthropometric and biochemical parameters in 25 obese patients with symptoms of metabolic syndrome, aged 30–60 years, with a BMI ≥ 30 kg/m^2^. Patients were assigned to two groups: the placebo group (following a diet combined with physical activity) and the study group (following the same diet and physical activity regimen, along with taking 250 mg/day of resveratrol for three months). The results of the study showed that supplementation with resveratrol reduced the total cholesterol (from 221.0 ± 48.6 to 192.1 ± 43.9 mg/dL), high density lipoprotein (HDL) (from 42.7 ± 7.6 to 48.1 ± 6.2 mg/dL), cholesterol, very low density lipoprotein cholesterol (from 49.3 ± 25.6 to 39.1 ± 14.3 mg/dL), creatinine (from 1.0 ± 0.1 to 0.9 ± 0.2 mg/dL), and serum albumin (from 4.8 ± 0.6 to 4.0 ± 0.4 mg/dL). However, the small sample size and combined intervention (diet, physical activity, and supplementation) make it difficult to isolate the specific effect of resveratrol [99]. A study carried out by Heebøll et al. [100] was also conducted in 26 overweight and fatty liver subjects taking high doses of resveratrol (1500 mg daily) for six months. Patients treated with resveratrol showed a 3.8% decrease in liver lipid content, but resveratrol supplementation showed no improvement in insulin sensitivity or markers of metabolic syndrome, except for a transient decrease in blood pressure. Slight improvement in liver parameters was observed, indicating inconsistent findings across studies, possibly due to differences in dose and study populations [100]. In a study by Simental-Mendía et al. [101], the use of resveratrol by 71 subjects aged 20 to 65 years with newly diagnosed dyslipidemia (dose of 100 mg/day) for two months showed significant reductions in total cholesterol (220.6 ± 37.4 vs. 201.4 ± 34.4 mg/dL) and triglycerides (166.7 ± 68.5 vs. 133.4 ± 55.3 mg/dL) compared to the placebo group [101]. However, another study by Zhou et al. [102] on the effect of resveratrol supplementation on lipid profile and other metabolic markers in subjects with dyslipidemia did not show a significant improvement in lipid profile parameters in 168 subjects randomly assigned to resveratrol groups (total of 125 subjects) at doses of 100 mg/day (*n* = 41), 300 mg/day (*n* = 43), and 600 mg/day (*n* = 41) compared to the placebo groups (*n* = 43). Anthropometric and biochemical parameters were analyzed at the start of the study and after 4 and 8 weeks. Only a significant decrease in serum uric acid levels was observed after 8 weeks in the groups taking 300 mg/day and 600 mg/day compared to the placebo. Resveratrol supplementation for 8 weeks did not improve the metabolic markers in subjects with dyslipidemia, further highlighting inconsistencies in the available evidence [102]. As in the meta-analysis of Li et al. [103] investigating the effect of resveratrol supplementation on lipid profile parameters in humans, it was observed that resveratrol supplement reduced triglycerides (weighted mean difference: −0.14 mg/dL, *p* = 0.001) and the total cholesterol (weighted mean difference: −0.20 mg/dL, *p* = 0.001), with the exception of HDL cholesterol and LDL cholesterol, but it should be noted that these decreases on a weighted average basis do not significantly affect lipid profile parameters and are of no clinical significance. Therefore, resveratrol supplements can only be used to support therapeutic activities in the treatment of dyslipidemia [103].

Resveratrol supplementation has also been investigated in glycemic control. The study by Mahjabeen et al. [104] aimed to determine the effect of resveratrol supplements on glucose metabolism, inflammation, oxidative stress, and inflammation in patients with type 2 diabetes taking oral hypoglycemic drugs. Patients with diabetes received resveratrol 200 mg once daily (*n* = 55) and the placebo (*n* = 55), cellulosic capsules for 24 weeks. The 24-week supplementation with resveratrol resulted in significant decreases in plasma glucose (7.56%), HbA1c (6.31%), insulin (9.96%), HOMA-IR (17.96%), C-reactive protein, tumor necrosis factor alpha, and interleukin-6. A reduction in HbA1c of approximately 0.5–0.7% is generally considered clinically meaningful in diabetes management (per ADA guidelines). The reported 6.31% reduction in HbA1c exceeds this threshold, indicating potential benefit, especially as an adjunct therapy. However, potential confounding from concomitant pharmacotherapy and lack of long-term follow-up should be considered. While resveratrol may have adjunctive potential, its clinical efficacy in glycemic control remains insufficiently established [104]. The meta-analysis by Gu et al. [105], which included 19 studies involving 1151 patients with type 2 diabetes—584 patients treated with resveratrol and 567 patients treated with placebo—indicated that high doses of resveratrol (≥1000 mg) lowered the fasting blood glucose levels (weighted mean difference: −18.76 mg/dL). Additionally, resveratrol reduced both systolic and diastolic blood pressure. However, no significant improvements were observed in triglycerides, HDL cholesterol, or waist circumference in patients with type 2 diabetes. Meta-analyses indicate that higher doses may reduce fasting glucose and blood pressure, but effects on other metabolic parameters remain inconsistent or negligible. Resveratrol is not currently recommended as standard therapy for glycemic control, with variability across studies and limited effects. However, these findings suggest that resveratrol is not recommended as a standard therapy but may be considered as a potential adjunct, pending further high-quality, standardized clinical trials. The current body of evidence is characterized by inconsistency, small to moderate effect sizes, and substantial heterogeneity across studies, which collectively limit the ability to draw robust and definitive clinical conclusions [105]. No sensitivity analyses were conducted due to the heterogeneity of the included studies. The risk of reporting bias was assessed qualitatively for each synthesis. Variability in reported outcomes, selective outcome reporting, and incomplete data in some studies suggest a potential risk of reporting bias, which may affect the interpretation of the findings.

## 5. Adverse Effects of Antioxidant Supplementation

Although numerous clinical studies have shown the beneficial effects of antioxidant supplementation, particularly anthocyanins, curcumin, and resveratrol, on weight control, metabolic parameters, and glycemic regulation, the potential adverse effects, contraindications, and long-term safety of these compounds are still not sufficiently discussed. However, the observed effects are not entirely consistent across studies, which may be explained by differences in study design, population characteristics, and intervention protocols (Table 5).

Several limitations of the included evidence should be considered. The studies varied substantially in terms of sample size, study design, duration of intervention, and dosage of antioxidants. Additionally, some studies reported limited outcome data, which may affect the robustness of the conclusions. Among the possible adverse effects, hepatotoxicity was noted, for example, in the study by Hellmann et al. [100], where minor changes in liver enzyme activity, especially γ-glutamyl transferase, were observed during a six-week curcumin supplementation in obese individuals. Additionally, mild gastrointestinal disturbances such as nausea, diarrhea, or abdominal discomfort have been reported in association with supplementation with curcumin, anthocyanins, and resveratrol. Furthermore, the metabolic response to supplementation is variable; large doses or long-term use do not always lead to the expected improvements in metabolic parameters. For example, long-term supplementation of resveratrol (1500 mg/day) in individuals with metabolic syndrome did not show significant improvement in insulin sensitivity or anthropometric indicators [100]. Similarly, research on anthocyanins did not confirm their effect on CRP, IL-6, or other glycemic markers after long-term use. It is also worth noting that resveratrol may interact with drugs metabolized by cytochrome P450 enzymes, as it can modulate CYP enzyme activity, as well as with anticoagulants and antiplatelet drugs, potentially altering their pharmacological effects [66]. Patients with metabolic disorders (e.g., obesity, diabetes, dyslipidemia) often exhibit altered absorption, distribution, metabolism, and excretion (ADME) of antioxidants. For example, obesity can affect the volume of distribution and hepatic metabolism, leading to different plasma levels compared to healthy individuals. Studies show that patients with metabolic diseases typically have higher baseline oxidative stress and inflammation, which may modify the efficacy of antioxidants. The antioxidant capacity and response to supplementation can differ markedly from healthy subjects. In addition, inter-individual variability in dietary patterns, lifestyle behaviors (e.g., physical activity and smoking), baseline metabolic status, and concurrent pharmacotherapy may further confound observed outcomes and contribute to heterogeneity across clinical studies. While study selection and data extraction were performed independently, the process may still be subject to subjective interpretation. Furthermore, the absence of a meta-analysis limits the ability to provide pooled estimates of effect. Gut microbiota, which influences polyphenol metabolism and bioavailability, is often dysbiotic in metabolic disorders, affecting the biotransformation and absorption of antioxidants like anthocyanins and curcumin. The pathophysiology of metabolic disorders may alter cellular responses to antioxidants, impacting their efficacy. For instance, insulin resistance may impair cellular uptake or signaling pathways influenced by antioxidants. Doses effective in healthy volunteers may be insufficient or unsafe in patients due to differences in pharmacokinetics and disease state, necessitating tailored dosing strategies and longer treatment durations (Table 6) [17].

The results of this review suggest that antioxidant supplementation may be a promising supportive strategy in the management of metabolic disorders. In light of the current data, future studies should focus on high-quality, long-term, randomized controlled trials to assess the safety and optimal dosing of antioxidant supplements. Despite the promising metabolic benefits, doctors should consider the potential side effects, contraindications, and regulatory limitations. The long-term safety of these compounds remains inadequately understood, which underscores the need for careful monitoring and further research in this area.

## 6. Conclusions

This review demonstrates the potential beneficial effects of antioxidants in the prevention and management of metabolic diseases such as obesity, diabetes, and dyslipidemia. The results of clinical studies indicate that antioxidant compounds, including anthocyanins, curcumin, and resveratrol, exhibit promising biological activity. However, their effects are generally modest and do not replace standard pharmacological treatment. Nevertheless, the current evidence is limited by the lack of long-term clinical trials, substantial heterogeneity among studies, and important pharmacokinetic constraints, including low bioavailability and rapid metabolism of these compounds. To maximize the effectiveness of antioxidant-based strategies, further research is needed to determine optimal dosage, bioavailability, and long-term safety in the therapy of metabolic disorders. Potential side effects of their use should also be identified, leading to better outcomes in the treatment of metabolic disorders. Additionally, future studies should focus on identifying specific patient populations that may benefit most from antioxidant supplementation and on understanding the mechanisms underlying their effects. Based on the evidence included in this review, the clinical findings highlight the potential role of selected dietary polyphenols (anthocyanins, curcumin, and resveratrol) as a complementary approach in the prevention and management of metabolic disorders. The present work provides a comparative synthesis of these compounds, integrating clinical outcomes with pharmacokinetic limitations and study heterogeneity, allowing for an assessment of their effects across major metabolic outcomes, including glycemic control, lipid profile, and inflammatory markers. The available evidence indicates that these compounds exert only modest effects in clinical settings, and their efficacy is strongly influenced by formulation, dosage, population characteristics, and bioavailability limitations. Importantly, the analysis highlights that current knowledge remains insufficient for translation into standardized clinical recommendations. Their role should be considered supportive rather than primary, and should be used in combination with established lifestyle practices and pharmacological interventions. Key gaps include the lack of long-term randomized controlled trials, limited head-to-head comparisons, and insufficient characterization of responsive patient subgroups. Addressing these limitations is essential to determine whether the observed biochemical effects translate into clinically meaningful benefits in metabolic disease management.

## Figures and Tables

**Table 1:** Study characteristics.

The study population	Patients with metabolic disorders, such as type 2 diabetes, obesity, insulin resistance, and dyslipidemia (number of participants from several dozen to several hundred; adult age, most often 30–65 years)
Intervention	Administering antioxidants such as vitamin C, vitamin E, resveratrol, curcumin, or combinations of these supplements, often in comparison to a placebo or standard treatment
Duration of the study	Typically, 4 to 24 weeks, with some studies lasting longer than 6 months
Main results	Improvement of metabolic parameters, such as fasting glucose levels, insulin, HOMA-IR index, lipid profiles (LDL, HDL, triglycerides), and reduction in inflammation and oxidative stress markers
Conclusions	Antioxidants may have a positive effect on improving metabolic parameters and reducing oxidative stress, suggesting their potential role as a supplement to therapy in metabolic disorders. However, further research is needed to determine the optimal dosage and long-term effects

**Table 2:** Clinical trial results of anthocyanin supplementation.

Author Reference	Study Design	Population (*n*)	Intervention	Duration	Main Results	Key Limitations	Heterogeneity Factors
Bertoia et al. [59]	Prospective cohort studies (3 cohorts)	124,086	FFQ flavonoid intake (224–247 mg/day)	Long-term	Lower weight gain over 4-year periods	Observational design; self-reported intake; confounding	Dietary variability; non-randomized
Park et al. [60]	RCT	>300	≤300 mg/day ≥4 weeks	Short-term	Lower BMI and body weight	Short duration; small trials	Dose; duration; populations
Cremonini et al. [61]	RCT (crossover study)	25	320 mg (berry extracts)	Acute	↓ postprandial inflammation	Very small sample; short-term	Study design; intervention type
Curtis et al. [62]	RCT	N/A	Blueberries (1 cup/day)	6 months	↑ HDL, improved vascular function	Limited metabolic endpoints	Dose; intervention form
Zhang et al. [52]	RCT	169	40–320 mg/day	6–12 weeks	↓ IL-6, TNF-α	Moderate duration	Dose-dependent effects
Es-tevez-Santiago et al. [62]	RCT	N/A	60 mg/day	8 months	No effect on CRP, IL-6	Null findings	Population differences
Guo et al. [63]	Prospective cohort studies	>200,000	Dietary intake (berry)	Long-term	↓ diabetes risk	Observational evidence	Diet variability
Fallah et al. [64]	RCT	N/A	>300 mg/day >8 weeks	Variable	↓ fasting blood glucose, HbA1c, HOMA-IR	Study heterogeneity	Dose; duration
Yang et al. [65]	RCT	N/A	320 mg/day	12 weeks	↓ HbA1c (~0.3–0.5%), ↓ LDL, ↓ ApoA, ApoB	Moderate sample size	Population; baseline status

↑ increase, ↓ decrease.

**Table 3:** Clinical trial results of curcumin supplementation.

Author (Reference)	Study Design	Population (*n*)	Duration	Main Results	Key Limitations	Heterogeneity Factors
Dehzad et al. [77]	Meta-analysis (60 RCTs)	3691	Variable	↓ Body weight (~0.82 kg), ↓ BMI (~0.30 kg/m^2^), ↓ waist circumference (~1.31 cm), ↓ body fat %, ↓ leptin (~4.46 ng/mL), ↑ adiponectin. Statistically significant but modest clinical relevance	High heterogeneity	Dose; formulation; duration
Unhapipatpong et al. [78]	RCT	N/A	Not specified	↓ BMI (~0.24 kg/m^2^), ↓ body weight (~0.59 kg), ↓ waist circumference (~1.31 cm). Beneficial but small clinical effect	Missing duration; small sample	Population; follow-up
Sangouni et al. [79]	RCT	88	12 weeks	↓ Triglycerides (224.7 → 166.7 mg/dL), ↓ Total cholesterol (217.4 → 186.5 mg/dL), ↓ LDL-C (89.4 → 75.3 mg/dL), ↑ HDL-C (32.0 mg/dL → 42.0 mg/dL). No significant effect on BMI, body weight, fasting glucose	No glycemic effect	Intervention combinations
Hellmann et al. [80]	RCT	37	6 weeks	Slight, non-significant ↓ fasting glucose and triglycerides; significant ↓ GGT. No significant effect on liver fat content	Small sample; short duration	Population
Yaikwawong et al. [81]	RCT	272	Not specified	↓ Fasting glucose (123.65 → 115.49 mg/dL), ↓ insulin, ↓ HbA1c (6.28 → 6.12%), ↓ HOMA-IR, ↓ leptin, ↑ adiponectin, improved β-cell function. HbA1c reduction (~0.16%) below ≥0.5% clinical relevance threshold	Below clinical threshold	Dose; population
Karandish et al. [82]	RCT	84	90 days	↓ BMI (~1.2 kg/m^2^ in intervention groups), improved fasting glucose, ↓ HbA1c, insulin	Moderate sample	Combination therapy

↑ increase, ↓ decrease.

**Table 4:** Results of clinical trials of resveratrol in metabolic disorders.

Author (Reference)	Study Design	Population (*n*)	Dose/ Duration	Main Results	Key Limitations	Heterogeneity Factors
Mousavi et al. [96]	Meta-analysis (28 clinical trials)	28 RCTs	<500 mg/ ≥3 months	Significant ↓ weight (weighted mean difference: −0.51 kg, *p* = 0.02) and BMI (−0.17 kg/m^2^, *p* = 0.02)	Limited clinical relevance	Dose; duration
Tabrizi et al. [97]	Meta-analysis (36 RCT)	36 RCTs	Variable	Small ↓ body weight (weighted mean difference: −0.17 kg, *p* = 0.03), fat tissue (−0.32 kg, *p* = 0.03), reduction in BMI (−0.20 kg/m^2^, *p* = 0.01) and waist circumference (−0.42 kg, *p* = 0.001), and increase in lean tissue (1.21 kg, *p* < 0.001)	High heterogeneity	Population; dose
De Ligt et al. [98]	RCT (cross-over)	11	150 mg/ 30 days	↓ angiotensinogen 2, leptin	Very small sample; short duration	Population (men only)
Batista-Jorge et al. [99]	RCT	25	250 mg	Improvement in lipid profile, reduction in total cholesterol (from 221.0 ± 48.6 to 192.1 ± 43.9 mg/dL), serum creatinine (from 1.0 ± 0.1 to 0.9 ± 0.2 mg/dL) and albumin (from 4.8 ± 0.6 to 4.0 ± 0.4 mg/dL), increase in HDL cholesterol (from 42.7 ± 7.6 to 48.1 ± 6.2 mg/dL)	Small sample	Combined interventions
Heebøll et al. [100]	RCT	26	1500 mg/ 6 months	No metabolic improvement	High dose variability	Population
Simental-Mendía et al. [101]	RCT	71	100 mg/ 2 months	↓ total cholesterol (220.6 ± 37.4 vs. 201.4 ± 34.4 mg/dL) and triglycerides (166.7 ± 68.5 vs. 133.4 ± 55.3 mg/dL)	Short duration	Dose
Zhou et al. [102]	RCT	168	100–600 mg/8 weeks	No significant effects	Not clinically meaningful	Dose variability
Li et al. [103]	Meta-analysis (21 clinical trials)	21 trials	Variable	Improved triglycerides (weighted mean difference: −0.14 mg/dL, *p* = 0.001), total cholesterol (weighted mean difference: −0.20 mg/dL, *p* = 0.001), except HDL cholesterol and LDL cholesterol, but not clinically relevant	Concomitant therapy	Study variability
Mahjabeen et al. [104]	RCT	10	200 mg/ 24 weeks	↓ glucose (7.56%), insulin (9.96%), HbA1c (6.31%), HOMA-IR (17.96%)	No lipid effect	Population
Gu et al. [105]	Meta-analysis	1151	≥1000 mg	↓ fasting glucose levels (weighted mean difference: −18.76 mg/dL), no change in the improvement of systolic and systolic blood pressure, waist circumference, lipid profile parameters		Dose differences

↓ decrease.

**Table 5:** Assessment of study quality and risk of bias for antioxidant supplementation (anthocyanins, curcumin, resveratrol) in metabolic disorders.

Supplement	Study (Year)	Study Design	Sample Size (*n*)	Duration	Risk of Bias (Cochrane RoB)	Evidence Quality (GRADE)	Key Limitations	Sources of Heterogeneity
Anthocyanins	Bertoia et al. [59]	Cohort	124,086	Long-term	Moderate (prospective cohort, self-reported dietary intake)	Moderate	No randomization, risk of recall bias, observational results	Dietary variability; non-randomized design
Anthocyanins	Park et al. [60]	Meta-analysis of RCTs	>300	Short-term	Low to moderate (short-term RCTs)	High	Short intervention period, small sample in included trials	Dose; duration; population differences
Anthocyanins	Cremonini et al. [61]	RCT cross-over	25	Short-term	Moderate (small sample, short duration)	Low	Very small sample, limited statistical power	Study design; intervention duration
Curcumin	Dehzad et al. [77]	Meta-analysis of RCTs	3691	Variable	Low to moderate	High	Large sample, heterogeneity in doses and intervention duration	Dose; formulation; duration
Curcumin	Unhapipatpong et al. [78]	RCT	N/A	Short-term	Low	Moderate	Small sample, limited follow-up duration	Population; duration
Curcumin	Sangouni et al. [79]	RCT	88	Short-term	Moderate	Moderate	Small group, short-term, no effect on glycemia	Outcome variability
Resveratrol	Mousavi et al. [96]	Meta-analysis	28 RCTs	≥3 months	Low to moderate	High	Short intervention period, different doses <500 mg	Dose (<500 mg); population differences
Resveratrol	Tabrizi et al. [97]	Meta-analysis	36 RCTs	Variable	Low to moderate	High	Heterogeneity in doses and populations	Dose; duration; baseline metabolic status
Resveratrol	De Ligt et al. [98]	RCT cross-over	11	30 days	High	Low	Very small sample, only men, short duration	Population; intervention length

**Table 6:** Biochemical characteristics and pharmacokinetic properties of antioxidant compounds (anthocyanins, curcumin, resveratrol).

Biochemical Class	Compound	Absorption	Bioavailability	Metabolism	Elimination	Key Limitation	Translational Relevance
Polyphenols	Resveratrol	Moderate; rapid absorption	Low (~1%) due to rapid metabolism	Phase II conjugation (glucuronidation, sulfation)	Feces, urine	Low systemic levels	Limited concordance between in vitro activity and in vivo exposure; formulation-dependent; extensive first-pass metabolism limits plasma levels; bioavailability varies with formulation
Curcumin	Curcumin	Poor; low absorption	Very low (~1%)	Extensive conjugation (glucuronidation, sulfation)	Bile, urine	Low absorption; rapid metabolism	Enhanced bioavailability with formulations; e.g., nanoparticles, adjuvants
Anthocyanins	Cyanidin-3-glucoside	Rapid; pH-sensitive stability	Low (~1–2%)	Rapid metabolism to phenolic acids	Urine	Instability; short half-life	Short half-life; Effects influenced by food matrix and metabolic transformation

## Data Availability

Data supporting the findings of this study are available within the article and its Supplementary Materials.

## Supplementary Materials

The following supporting information can be downloaded at: https://www.mdpi.com/article/10.3390/molecules31111837/s1, PRISMA 2020 Checklist [15].

## Abbreviations

The following abbreviations are used in this manuscript:

ACE2	Angiotensin-converting enzyme 2
ADA	American Diabetes Association
cis-/trans	Cis- and trans-isomers
COX-2	Cyclooxygenase-2
CYP	Cytochrome
DNA	Deoxyribonucleic acid
ESC	European Society of Cardiology
FFAs	Free fatty acids
H3K56ac	Histone H3 lysine 56 acetylation
HbA1c	Glycated hemoglobin
HDL	High-density lipoprotein levels
HOMA-IR	Homeostatic Model Assessment-Insulin Resistance
IGT	Impaired glucose tolerance
IL-1	Interleukin-1
IL-6	Interleukin-6
iNOS	Inducible nitric oxide synthase
IRS	Insulin receptor substrate
LDL	Low-density lipoprotein
MDA	Malondialdehyde
NF-κB (NF-KB)	Nuclear factor kappa-light-chain-enhancer of activated B cells
Nrf2	Nuclear factor erythroid 2–related factor 2
OH	Hydroxyl group/hydroxyl radical (•OH)
RINm5F	Rat insulinoma 5F
ROS	Reactive oxygen species
SIRT1	Sirtuin 1
SOD	Superoxide dismutase
TNF-α	Tumor necrosis factor

## References

[1] Khutami C., Sumiwi S.A., KhairulIkram N.K., Muchtaridi M. (2022). Effect of antioxidants from natural products on obesity, dyslipidemia, diabetes and their molecular signaling mechanism. Int. J. Mol. Sci..

[2] Ostrowska O.L., Bogdański P., Mamcarz A. (2021). Obesity and Its Complications: Practical Diagnostic and Therapeutic Recommendations.

[3] Spahis S., Borys J.M., Levy E. (2017). Metabolic Syndrome as a Multifaceted Risk Factor for Oxidative Stress. Antioxid. Redox Signal..

[4] Torres S., Fabersani E., Marquez A., Gauffin-Cano P. (2019). Adipose tissue inflammation and metabolic syndrome. The proactive role of probiotics. Eur. J. Nutr..

[5] Holmstrom L., Junttila J., Chugh S.S. (2024). Sudden Death in Obesity: Mechanisms and Management. J. Am. Coll. Cardiol..

[6] Mayoral L.P., Andrade G.M., Mayoral E.P., Huerta T.H., Canseco S.P., Canales F.J.R., Cabrera-Fuentes H.A., Cruz M.M., Santiago A.D.P., Alpuche J.J. (2020). Obesity Subtypes, Related Biomarkers & Heterogeneity. Indian J. Med. Res..

[7] Lee K.X., Quek K.F., Ramadas A. (2023). Dietary and Lifestyle Risk Factors of Obesity Among Young Adults: A Scoping Review of Observational Studies. Curr. Nutr. Rep..

[8] Apovian C.M. (2016). Obesity: Definition, comorbidities, causes, and burden. Am. J. Manag. Care.

[9] Furukawa S., Fujita T., Shimabukuro M., Iwaki M., Yamada Y., Nakajima Y., Nakayama O., Makishima M., Matsuda M., Shimomura I. (2017). Increased oxidative stress in obesity and its effect on metabolic syndrome. J. Clin. Investig..

[10] Martemucci G., Portincasa P., Centonze V., Mariano M., Khalil M., D’ALessandro A.G. (2023). Prevention of Oxidative Stress and Diseases by Antioxidant Supplementation. Med. Chem..

[11] Xue R., Li Q., Geng Y., Wang H., Wang F., Zheng S. (2021). Abdominal obesity and risk of CVD: A dose-response meta-analysis of thirty-one prospective studies. Br. J. Nutr..

[12] Nussbaumerova B., Rosolova H. (2023). Obesity and Dyslipidemia. Curr. Atheroscler. Rep..

[13] Vekic J., Zeljkovic A., Stefanovic A., Jelic-Ivanovic Z., Spasojevic-Kalimanovska V. (2019). Obesity and dyslipidemia. Metabolism.

[14] Sun H., Saeedi P., Karuranga S., Pinkepank M., Ogurtsova K., Duncan B.B., Stein C., Basit A., Chan J.C., Mbanya J.C. (2022). Diabetes Atlas: Global, regional and country-level diabetes prevalence estimates for 2021 and projections for 2045. Diabetes Res. Clin. Pract..

[15] Page M.J., McKenzie J.E., Bossuyt P.M., Boutron I., Hoffmann T.C., Mulrow C.D., Shamseer L., Tetzlaff J.M., Akl E.A., Brennan S.E. (2021). The PRISMA 2020 statement: An updated guideline for reporting systematic reviews. BMJ.

[16] Senoner T., Dichtl W. (2019). Oxidative Stress in Cardiovascular Diseases: Still a Therapeutic Target?. Nutrients.

[17] He L., He T., Farrar S., Ji L., Liu T., Ma X. (2017). Antioxidants Maintain Cellular Redox Homeostasis by Elimination of Reactive Oxygen Species. Cell Physiol. Biochem..

[18] Gonzalez A., Simon F., Achiardi O., Vilos C., Cabrera D., Cabello-Verrugio C. (2021). The Critical Role of Oxidative Stress in Sarcopenic Obesity. Oxidative Med. Cell. Longev..

[19] Pérez-Torres I., Castrejón-Téllez V., Soto M.E., Rubio-Ruiz M.E., Manzano-Pech L., Guarner-Lans V. (2021). Oxidative Stress, Plant Natural Antioxidants, and Obesity. Int. J. Mol. Sci..

[20] Zielinska-Blizniewska H., Sitarek P., Merecz-Sadowska A., Malinowska K., Zajdel K., Jablonska M., Sliwinski T., Zajdel R. (2019). Plant extracts and reactive forms of oxygen as two counteracting agents with anti-obesity and anti-obesity properties. Int. J. Mol. Sci..

[21] Márquez Álvarez C.M., Hernández-Cruz E.Y., Pedraza-Chaverri J. (2023). Oxidative stress in animal models of obesity caused by hypercaloric diets: A systematic review. Life Sci..

[22] De Mello A.H., Costa A.B., Engel J.D.G., Rezin G.T. (2018). Mitochondrial dysfunction in obesity. Life Sci..

[23] Bhatti J.S., Bhatti G.K., Reddy P.H. (2017). Mitochondrial dysfunction and oxidative stress in metabolic disorders—A step towards mitochondria based therapeutic strategies. Biochim. Biophys. Acta Mol. Basis Dis..

[24] Luc K., Schramm-Luc A., Guzik T.J., Mikolajczyk T.P. (2019). Oxidative stress and inflammatory markers in prediabetes and diabetes. J. Physiol. Pharmacol..

[25] Na I.J., Park J.S., Park S.B. (2019). Relationship between abdominal obesity and oxidative stress in Korean adults. Korean J. Fam. Med..

[26] Braga T., Kraemer-Aguiar L.G., Docherty N.G., Le Roux C.W. (2019). Treating prediabetes: Why and how should we do it?. Minerva Med..

[27] Khan R.M.M., Chua Z.J.Y., Tan J.C., Yang Y., Liao Z., Zhao Y. (2019). From Pre-Diabetes to Diabetes: Diagnosis, Treatments and Translational Research. Medicina.

[28] Skyler J.S., Bakris G.L., Bonifacio E., Darsow T., Eckel R.H., Groop L., Groop P.-H., Handelsman Y., Insel R.A., Mathieu C. (2017). Differentiation of Diabetes by Pathophysiology, Natural History, and Prognosis. Diabetes.

[29] Lee S.H., Park S.Y., Choi C.S. (2022). Insulin Resistance: From Mechanisms to Therapeutic Strategies. Diabetes Metab. J..

[30] González P., Lozano P., Ros G., Solano F. (2023). Hyperglycemia and Oxidative Stress: An Integral, Updated and Critical Overview of Their Metabolic Interconnections. Int. J. Mol. Sci..

[31] Newsholme P., Keane K.N., Carlessi R., Cruzat V. (2019). Oxidative stress pathways in pancreatic β-cells and insulin-sensitive cells and tissues: Importance to cell metabolism, function, and dysfunction. Am. J. Physiol. Cell Physiol..

[32] Agarwal A., Hegde A., Yadav C., Ahmad A., Manjrekar P.A., Srikantiah R.M. (2016). Assessment of oxidative stress and inflammation in prediabetes-A hospital based cross-sectional study. Diabetes Metab. Syndr..

[33] Noras K., Rusak E., Jarosz-Chobot P. (2021). The Problem of Abnormal Body Weight and Dyslipidemia as Risk Factors for Cardiovascular Diseases in Children and Adolescents with Type 1 Diabetes. J. Diabetes Res..

[34] Du Y., Lv Y., Zha W., Hong X., Luo Q. (2020). Effect of coffee consumption on dyslipidemia: A meta-analysis of randomized controlled trials. Nutr. Metab. Cardiovasc. Dis..

[35] Chopra A.K. (2024). Dietary management of dyslipidemia. Indian Heart J..

[36] Vázquez-Oliva G., Zamora A., Ramos R., Subirana I., Grau M., Dégano I.R., Muñoz D., Fitó M., Elosua R., Marrugat J. (2018). Valor predictivo de la albúminaplasmática, la vitamina D y las apolipoproteínas A y B comobiomarcadores de riesgocoronarioenelestudio REGICOR. Rev. Española Cardiol..

[37] Su X., Peng D. (2020). The exchangeable apolipoproteins in lipid metabolism and obesity. Clin. Chim. Acta.

[38] Uchmanowicz I. (2020). Oxidative Stress, Frailty and Cardiovascular Diseases: Current Evidence. Adv. Exp. Med. Biol..

[39] El Hadri K., Smith R., Duplus E., El Amri C. (2021). Inflammation, Oxidative Stress, Senescence in Atherosclerosis: Thioredoxine-1 as an Emerging Therapeutic Target. Int. J. Mol. Sci..

[40] Incalza M.A., D’Oria R., Natalicchio A., Perrini S., Laviola L., Giorgino F. (2018). Oxidative stress and reactive oxygen species in endothelial dysfunction associated with cardiovascular and metabolic diseases. Vasc. Pharmacol..

[41] Hęś M., Dziedzic K., Górecka D., Jędrusek-Golińska A., Gujska E. (2019). Natural Sources of Antioxidants—A Review. Plant Foods Hum. Nutr..

[42] Sarkate A.P., Jambhorkar V.S., Sakhale B.K. (2020). Natural Food Antioxidants BT—Plant Antioxidants and Health.

[43] Apak R., Calokerinos A., Gorinstein S., Segundo M.A., Hibbert D.B., Gülçin I., Çekiç S.D., Güçlü K., Özyürek M., Çelik S.E. (2022). Methods to evaluate the scavenging activity of antioxidants toward reactive oxygen and nitrogen species (IUPAC Technical Report). Pure Appl. Chem..

[44] Chugh B., Kamal-Eldin A. (2020). Bioactive compounds produced by probiotics in food products. Curr. Opin. Food Sci..

[45] Toydemir G., Gultekin Subasi B., Hall R.D., Beekwilder J., Boyacioglu D., Capanoglu E. (2022). Effect of food processing on antioxidants, their bioavailability and potential relevance to human health. Food Chem. X.

[46] Nani A., Murtaza B., Khan A.S., Khan N.A., Hichami A. (2021). Antioxidant and Anti-Inflammatory Potential of Polyphenols Contained in Mediterranean Diet in Obesity: Molecular Mechanisms. Molecules.

[47] Sivamaruthi B.S., Kesika P., Chaiyasut C. (2018). Anthocyanins in Thai rice varieties: Distribution and pharmacological significance. Int. Food Res. J..

[48] Zhang J., Celli G.B., Brooks M.S., Brooks M.S.-L., Celli G.B. (2019). Natural sources of anthocyanins. Anthocyanins from Natural Sources: Exploiting Targeted Delivery for Improved Health.

[49] Liang A., Leonard W., Beasley J.T., Fang Z., Zhang P., Ranadheera C.S. (2024). Anthocyanins-gut microbiota-health axis: A review. Crit. Rev. Food Sci. Nutr..

[50] Khoo H.E., Azlan A., Tang S.T., Lim S.M. (2017). Anthocyanidins and anthocyanins: Colored pigments as food, pharmaceutical ingredients, and the potential health benefits. Food Nutr. Res..

[51] Sivamaruthi B.S., Kesika P., Chaiyasut C. (2020). The Influence of Supplementation of Anthocyanins on Obesity-Associated Comorbidities: A Concise Review. Foods.

[52] Zhang H., Xu Z., Zhao H., Wang X., Pang J., Li Q., Yang Y., Ling W. (2020). Anthocyanin supplementation improves anti-oxidative and anti-inflammatory capacity in a dose-response manner in subjects with dyslipidemia. Redox Biol..

[53] Xu L., Tian Z., Chen H., Zhao Y., Yang Y. (2021). Anthocyanins, Anthocyanin-Rich Berries, and Cardiovascular Risks: Systematic Review and Meta-Analysis of 44 Randomized Controlled Trials and 15 Prospective Cohort Studies. Front. Nutr..

[54] Liu X., Ge K., Shi H., Yao Z., Zhang Z. (2025). Effects of anthocyanins on human health: An umbrella review of systematic reviews and meta-analyses. Food Funct..

[55] Fallah A.A., Sarmast E., Jafari T. (2020). Effect of dietary anthocyanins on biomarkers of glycemic control and glucose metabolism: A systematic review and meta-analysis of randomized clinical trials. Food Res. Int..

[56] De Oliveira M.S., Pellenz F.M., de Souza B.M., Crispim D. (2022). Blueberry Consumption and Changes in Obesity and Diabetes Mellitus Outcomes: A Systematic Review. Metabolites.

[57] Mostafa H., Behrendt I., Meroño T., González-Domínguez R., Fasshauer M., Rudloff S., Andres-Lacueva C., Kuntz S. (2023). Plasma anthocyanins and their metabolites reduce in vitro migration of pancreatic cancer cells, PANC-1, in a FAK- and NF-kB dependent manner: Results from the ATTACH-study a randomized, controlled, crossover trial in healthy subjects. Biomed. Pharmacother..

[58] Brum I.S.D.C., Mafra D., Moreira L.S.G., Teixeira K.T.R., Stockler-Pinto M.B., Cardozo L.F.M.F., Borges N.A. (2022). Consumption of oils and anthocyanins may positively modulate PPAR-γ expression in chronic noncommunicable diseases: A systematic review. Nutr. Res..

[59] Bertoia M.L., Rimm E.B., Mukamal K.J., Hu F.B., Willett W.C., Cassidy A. (2016). Dietary flavonoid intake and weight maintenance: Three prospective cohorts of 124 086 US men and women followed for up to 24 years. BMJ.

[60] Park S., Choi M., Lee M. (2021). Effects of Anthocyanin Supplementation on Reduction of Obesity Criteria: A Systematic Review and Meta-Analysis of Randomized Controlled Trials. Nutrients.

[61] Cremonini E., Daveri E., Iglesias D.E., Kang J., Wang Z., Gray R., Mastaloudis A., Kay C.D., Hester S.N., Wood S.M. (2022). A randomized placebo-controlled cross-over study on the effects of anthocyanins on inflammatory and metabolic responses to a high-fat meal in healthy subjects. Redox Biol..

[62] Curtis P.J., van der Velpen V., Berends L., Jennings A., Feelisch M., Umpleby A.M., Evans M., O Fernandez B., Meiss M.S., Minnion M. (2019). Blueberries improve biomarkers of cardiometabolic function in participants with metabolic syndrome-results from a 6-month, double-blind, randomized controlled trial. Am. J. Clin. Nutr..

[63] Guo X., Yang B., Tan J., Jiang J., Li D. (2016). Associations of dietary intakes of anthocyanins and berry fruits with risk of type 2 diabetes mellitus: A systematic review and meta-analysis of prospective cohort studies. Eur. J. Clin. Nutr..

[64] Fallah A.A., Sarmast E., Fatehi P., Jafari T. (2020). Impact of dietary anthocyanins on systemic and vascular inflammation: Systematic review and meta-analysis on randomised clinical trials. Food Chem. Toxicol..

[65] Yang L., Ling W., Yang Y., Chen Y., Tian Z., Du Z., Chen J., Xie Y., Liu Z., Yang L. (2017). Role of Purified Anthocyanins in Improving Cardiometabolic Risk Factors in Chinese Men and Women with Prediabetes or Early Untreated Diabetes-A Randomized Controlled Trial. Nutrients.

[66] Estévez-Santiago R., Silván J.M., Can-Cauich C.A., Veses A.M., Alvarez-Acero I., Martinez-Bartolome M.A., San-Román R., Cámara M., Olmedilla-Alonso B., De Pascual-Teresa S. (2019). Lack of a synergistic effect on cardiometabolic and redox markers in a dietary supplementation with anthocyanins and xanthophylls in postmenopausal women. Nutrients.

[67] Nelson K.M., Dahlin J.L., Bisson J., Graham J., Pauli G.F., Walters M.A. (2017). The Essential Medicinal Chemistry of Curcumin. J. Med. Chem..

[68] Heidari H., Bagherniya M., Majeed M., Sathyapalan T., Jamialahmadi T., Sahebkar A. (2023). Curcumin-piperine co-supplementation and human health: A comprehensive review of preclinical and clinical studies. Phytother. Res..

[69] Kotha R.R., Luthria D.L. (2019). Curcumin: Biological, Pharmaceutical, Nutraceutical, and Analytical Aspects. Molecules.

[70] Mohammadian K., Fakhar F., Keramat S., Stanek A. (2024). The Role of Antioxidants in the Treatment of Metabolic Dysfunction-Associated Fatty Liver Disease: A Systematic Review. Antioxidants.

[71] Hu M., Cai J.-Y., He Y., Chen K., Hao F., Kang J.S., Pan Y., Tie L., Li X.J. (2024). Protective effects of curcumin on desipramine-induced islet beta-cell damage via AKAP150/PKA/PP2B complex. Acta Pharmacol. Sin..

[72] Sarmiento-Ortega V.E., Moroni-González D., Diaz A., Brambila E., Treviño S. (2025). Curcumin treatment ameliorates hepatic insulin resistance induced by sub-chronic oral exposure to cadmium LOAEL dose via NF-κB and Nrf2 pathways. Biol. Trace Elem. Res..

[73] Shen J.D., Wei Y., Li Y.J., Qiao J.-Y., Li Y.C. (2017). Curcumin reverses the depressive-like behavior and insulin resistance induced by chronic mild stress. Metab. Brain Dis..

[74] Wang Y., Liu D., Li X., Liu Y., Wu Y. (2021). Antidepressants use and the risk of type 2 diabetes mellitus: A systematic review and meta-analysis. J. Affect. Disord..

[75] Qin S., Huang L., Gong J., Shen S., Huang J., Tang Y., Ren H., Hu H. (2018). Meta-analysis of randomized controlled trials of 4 weeks or longer suggest that curcumin may afford some protection against oxidative stress. Nutr. Res..

[76] Bianconi V., Pirro M., Moallem S.M.H., Majeed M., Bronzo P., D’aBbondanza M., Jamialahmadi T., Sahebkar A. (2021). The Multifaceted Actions of Curcumin in Obesity. Adv. Exp. Med. Biol..

[77] Dehzad M.J., Ghalandari H., Nouri M., Askarpour M. (2023). Effects of curcumin/turmeric supplementation on obesity indices and adipokines in adults: A grade-assessed systematic review and dose-response meta-analysis of randomized controlled trials. Phytother. Res..

[78] Unhapipatpong C., Polruang N., Shantavasinkul P.C., Julanon N., Numthavaj P., Thakkinstian A. (2023). The effect of curcumin supplementation on weight loss and anthropometric indices: An umbrella review and updated meta-analyses of randomized controlled trials. Am. J. Clin. Nutr..

[79] Sangouni A.A., Taghdir M., Mirahmadi J., Sepandi M., Parastouei K. (2022). Effects of curcumin and/or coenzyme Q10 supplementation on metabolic control in subjects with metabolic syndrome: A randomized clinical trial. Nutr. J..

[80] Hellmann P.H., Bagger J.I., Carlander K.R., Forman J., Chabanova E., Svenningsen J.S., Holst J.J., Gillum M.P., Vilsbøll T., Knop F.K. (2022). The effect of curcumin on hepatic fat content in individuals with obesity. Diabetes Obes. Metab..

[81] Yaikwawong M., Jansarikit L., Jirawatnotai S., Chuengsamarn S. (2024). Curcumin extract improves beta cell functions in obese patients with type 2 diabetes: A randomized controlled trial. Nutr. J..

[82] Karandish M., Mozaffari-Khosravi H., Mohammadi S.M., Cheraghian B., Azhdari M. (2021). The effect of curcumin and zinc co-supplementation on glycemic parameters in overweight or obese prediabetic subjects: A phase 2 randomized, placebo-controlled trial with a multi-arm, parallel-group design. Phytother. Res..

[83] Le P., Ayers G., Misra-Hebert A.D., Herzig S.J., Herman W.H., Shaker V.A., Rothberg M.B. (2022). Adherence to the American Diabetes Association’s Glycemic Goals in the Treatment of Diabetes Among Older Americans, 2001–2018. Diabetes Care.

[84] Galiniak S., Aebisher D., Bartusik-Aebisher D. (2019). Health benefits of resveratrol administration. Acta Biochim. Pol..

[85] Inchingolo A.D., Inchingolo A.M., Malcangi G., Avantario P., Azzollini D., Buongiorno S., Viapiano F., Campanelli M., Ciocia A.M., De Leonardis N. (2022). Effects of Resveratrol, Curcumin and Quercetin Supplementation on Bone Metabolism-A Systematic Review. Nutrients.

[86] Zhou D.D., Luo M., Huang S.Y., Saimaiti A., Shang A., Gan R.Y., Li H.B. (2021). Effects and Mechanisms of Resveratrol on Aging and Age-Related Diseases. Oxidative Med. Cell. Longev..

[87] Breuss J.M., Atanasov A.G., Uhrin P. (2019). Resveratrol and Its Effects on the Vascular System. Int. J. Mol. Sci..

[88] Huang D.D., Shi G., Jiang Y., Yao C., Zhu C. (2020). A review on the potential of Resveratrol in prevention and therapy of diabetes and diabetic complications. Biomed. Pharmacother..

[89] Barber T.M., Kabisch S., Randeva H.S., Pfeiffer A.F.H., Weickert M.O. (2022). Implications of Resveratrol in Obesity and Insulin Resistance: A State-of-the-Art Review. Nutrients.

[90] Abbasi Oshaghi E., Goodarzi M.T., Higgins V., Adeli K. (2017). Role of resveratrol in the management of insulin resistance and related conditions: Mechanism of action. Crit. Rev. Clin. Lab. Sci..

[91] Seyyedebrahimi S., Khodabandehloo H., Nasli Esfahani E., Meshkani R. (2018). The effects of resveratrol on markers of oxidative stress in patients with type 2 diabetes: A randomized, double-blind, placebo-controlled clinical trial. Acta Diabetol..

[92] Saldanha J.F., Leal V.O., Rizzetto F., Grimmer G.H., Ribeiro-Alves M., Daleprane J.B., Carraro-Eduardo J.C., Mafra D. (2016). Effects of Resveratrol Supplementation in Nrf2 and NF-κB Expressions in Nondialyzed Chronic Kidney Disease Patients: A Randomized, Double-Blind, Placebo-Controlled, Crossover Clinical Trial. J. Ren. Nutr..

[93] Brenjian S., Moini A., Yamini N., Kashani L., Faridmojtahedi M., Bahramrezaie M., Khodarahmian M., Amidi F. (2020). Resveratrol treatment in patients with polycystic ovary syndrome decreased pro-inflammatory and endoplasmic reticulum stress markers. Am. J. Reprod. Immunol..

[94] Zhou J., Yang D., Liu K., Hou L., Zhang W. (2019). Systematic review and meta-analysis of the protective effect of resveratrol on multiple organ injury induced by sepsis in animal models. Biomed. Rep..

[95] Najafi M., Nikpayam O., Tavakoli-Rouzbehani O.M., Papi S., Bioky A.A., Ahmadiani E.S., Sohrab G. (2021). A comprehensive insight into the potential effects of resveratrol supplementation on SIRT-1: A systematic review. Diabetes Metab. Syndr..

[96] Mousavi S.M., Milajerdi A., Sheikhi A., Kord-Varkaneh H., Feinle-Bisset C., Larijani B., Esmaillzadeh A. (2019). Resveratrol supplementation significantly influences obesity measures: A systematic review and dose-response meta-analysis of randomized controlled trials. Obes. Rev..

[97] Tabrizi R., Tamtaji O.R., Lankarani K.B., Akbari M., Dadgostar E., Dabbaghmanesh M.H., Kolahdooz F., Shamshirian A., Momen-Heravi M., Asemi Z. (2020). The effects of resveratrol intake on weight loss: A systematic review and meta-analysis of randomized controlled trials. Crit. Rev. Food Sci. Nutr..

[98] De Ligt M., Hesselink M.K.C., Jorgensen J., Hoebers N., Blaak E.E., Goossens G.H. (2021). Resveratrol supplementation reduces ACE2 expression in human adipose tissue. Adipocyte.

[99] Batista-Jorge G.C., Barcala-Jorge A.S., Silveira M.F., Lelis D., Andrade J., de Paula A., Guimarães A., Santos S. (2020). Oral resveratrol supplementation improves Metabolic Syndrome features in obese patients submitted to a lifestyle-changing program. Life Sci..

[100] Heebøll S., Kreuzfeldt M., Hamilton-Dutoit S., Poulsen M.K., Stødkilde-Jørgensen H., Møller H.J., Jessen N., Thorsen K., Hellberg Y.K., Pedersen S.B. (2016). Placebo-controlled, randomised clinical trial: High-dose resveratrol treatment for non-alcoholic fatty liver disease. Scand. J. Gastroenterol..

[101] Simental-Mendía L.E., Guerrero-Romero F. (2019). Effect of resveratrol supplementation on lipid profile in subjects with dyslipidemia: A randomized double-blind, placebo-controlled trial. Nutrition.

[102] Zhou Y., Zeng Y., Pan Z., Jin Y., Li Q., Pang J., Wang X., Chen Y., Yang Y., Ling W. (2023). A Randomized Trial on Resveratrol Supplement Affecting Lipid Profile and Other Metabolic Markers in Subjects with Dyslipidemia. Nutrients.

[103] Li Z., Liu S., Liu Q., Wang M., Haedi A.R., Zang S.S., Li J.-L. (2024). Efficacy of resveratrol supplementation on lipid profile parameters: An umbrella of meta-analysis. Prostaglandins Other Lipid Mediat..

[104] Mahjabeen W., Khan D.A., Mirza S.A. (2022). Role of resveratrol supplementation in regulation of glucose hemostasis, inflammation and oxidative stress in patients with diabetes mellitus type 2: A randomized, placebo-controlled trial. Complement. Ther. Med..

[105] Gu W., Geng J., Zhao H., Li X., Song G. (2022). Effects of Resveratrol on Metabolic Indicators in Patients with Type 2 Diabetes: A Systematic Review and Meta-Analysis. Int. J. Clin. Pract..

